# Faecal hsa-miR-7704 inhibits the growth and adhesion of *Bifidobacterium longum* by suppressing ProB and aggravates hepatic encephalopathy

**DOI:** 10.1038/s41522-024-00487-8

**Published:** 2024-02-24

**Authors:** Yuchong Wang, Yuyu Li, Longxian Lv, Liying Zhu, Liang Hong, Xueyao Wang, Yu Zhang, Xin Wang, Hongyan Diao

**Affiliations:** 1 State Key Laboratory for Diagnosis and Treatment of Infectious Diseases, National Clinical Research Center for Infectious Diseases, National Medical Center for Infectious Diseases, Collaborative Innovation Center for Diagnosis and Treatment of Infectious Diseases, The First Affiliated Hospital, Zhejiang University School of Medicine, Hangzhou, 310003 China; 2 State Key Laboratory for Managing Biotic and Chemical Threats to the Quality and Safety of Agro-products, Institute of Food Science, Zhejiang Academy of Agricultural Sciences, Hangzhou, 310021 China; 3 Jinan Microecological Biomedicine Shandong Laboratory, Jinan, Shandong Province China

**Keywords:** Microbiota, Clinical microbiology

## Abstract

Both gut microbiome and microRNAs (miRNAs) play a role in the development of hepatic encephalopathy (HE). However, the functional link between the microbiome and host-derived miRNAs in faeces remains poorly understood. In the present study, patients with HE had an altered gut microbiome and faecal miRNAs compared with patients with chronic hepatitis B. Transferring faeces and faecal miRNAs from patients with HE to the recipient mice aggravated thioacetamide-induced HE. Oral gavage of hsa-miR-7704, a host-derived miRNA highly enriched in faeces from patients with HE, aggravated HE in mice in a microbiome-dependent manner. Mechanistically, hsa-miR-7704 inhibited the growth and adhesion of *Bifidobacterium longum* by suppressing *proB*. *B. longum* and its metabolite acetate alleviated HE by inhibiting microglial activation and ammonia production. Our findings reveal the role of miRNA–microbiome axis in HE and suggest that faecal hsa-miR-7704 are potential regulators of HE progression.

## Introduction

Hepatic encephalopathy (HE) is a common and serious complication of liver dysfunction. It manifests as a wide spectrum of neuropsychiatric abnormalities, ranging from mild cognitive impairment to marked asterixis, disorientation, and coma^1^. HE due to hepatitis B virus (HBV)-related acute-on-chronic liver failure (HBV-ACLF) is common in China and is characterised by a high short-term mortality rate. HE imposes considerable clinical and economic burden and occurs due to a combination of pathophysiological mechanisms, including inflammation, oxidative stress, neurotoxins, increased circulating ammonia concentration, and impairment in the blood-brain barrier (BBB) permeability^2^.

Gut microbiota communicates with the brain through several channels, such as neuronal, endocrine, and immune-mediated pathways^3^. HE has been widely reported to be closely associated with gut microbiota alterations^4^. Current treatments targeting the gut microbiota include lactulose, broad-spectrum antibiotics, and reduction of protein ingestion, which remain the current standard of care for HE^5,6^. Another therapeutic strategy for HE is faecal microbiota transplantation, which involves the introduction of healthy donor microbiota into a recipient^7–10^. Therefore, exploring microbial therapies that may provide protection against HE is important.

MicroRNAs (miRNAs) are small non-coding RNAs that regulate gene expression. Recent studies have confirmed that faecal miRNAs shape the gut microbiome and influence disease progression^11–13^. In intestinal epithelial cells, miRNA deficiency is associated with exacerbated dextran sulfate sodium (DSS)-induced colitis^11^. A previous study showed that faecal miRNA from patients with multiple sclerosis alleviated disease by altering gut microbes^12^. Another report indicated that faeces from individuals who recovered from DSS-induced colitis were enriched with miRNAs exhibiting preventive effects^13^. These miRNAs exert their effects in a microbiome-dependent manner and affect the growth and metabolism of specific microbes. Nonetheless, whether miRNAs in faeces from patients with HE protect from disease progression and its underlying mechanisms remain unknown.

In the present study, we analysed the gut microbiome and miRNAs in HBV-ACLF cases with HE and compared them to those in chronic hepatitis B (CHB) cases. We established an experimental mouse model of HE using thioacetamide (TAA) and examined the alterations in the gut microbiome. Our findings reveal a novel mechanism by which faecal miRNAs modulate gut microbiota, further regulating the development of HE.

## Results

### Faecal microbiota composition differed between patients with HE and CHB

**Fig. 1:**
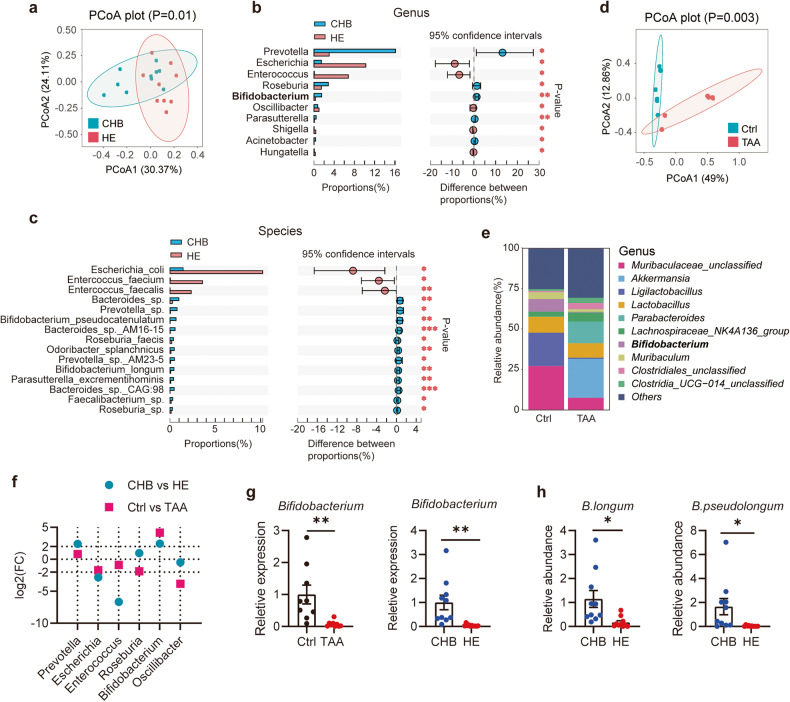
Changes in the faecal microbiota composition in HE. **a**–**c** Microbiome surveys based on metagenomics analysis were performed. Faeces were collected from patients with HE (*n* = 9) and patients with CHB (*n* = 10). **a** Principal-coordinate analysis (PCoA) based on Bray-Curtis distance. Statistical analyses of taxonomic and functional profiles (STAMP) were performed on genus- (**b**) and species- (**c**) levels. Microbiome analyses based on bacterial 16S rDNA sequence were performed. Mice were intraperitoneally injected with TAA for inducing HE, and faeces were collected before injection and at 48 h-post-injection. **d** PCoA based on Bray-Curtis distance. **e** Relative abundance of bacteria classified at a genus-level taxonomy. *n* = 6. **f** The fold change of bacteria according to metagenomics analysis and bacterial 16S rDNA sequence. **g** The relative abundance of *Bifidobacterium* in the faeces was quantified by Real-time qPCR analysis. **h** The relative abundance of *B. longum* and *B. pseudolongum* in the faeces of patients according to metagenomics analysis. Data are represented as mean ± SEM. *p < 0.05, **p < 0.01, determined by unpaired Student’s *t*-test (**g** and **h**).

The gut microbiome is essential for the gut-brain axis. This study recruited 9 patients with HBV-ACLF with HE and 10 with CHB; faecal samples were collected for analysis. Metagenomic analysis was performed to assess the faecal microbiota composition in patients with HE and CHB. For the microbial beta-diversity metric, we conducted a principal coordinate analysis based on the Bray–Curtis’s distance and found that the overall microbial structure differed between patients with HE and CHB (Fig. 1a). To identify the specific microbes influenced by HE progression, we compared them by performing statistical analyses of taxonomic and functional profiles (STAMP) at the genus (Fig. 1b) and species levels (Fig. 1c).

*Prevotella*, *Roseburia*, and *Bifidobacterium* were significantly lower in the HE group than in the CHB group. The abundance of *Escherichia*, *Enterococcus*, and *Oscillibacter* increased in the HE group (Fig. 1b). Our results at the species level revealed that the HE group had a higher relative abundance of *Escherichia coli*, *Enterococcus faecium*, and *Enterococcus faecalis* and a lower relative abundance of *Prevotella* sp, *Bifidobacterium pseudocatenulatum*, *Roseburia faecis*, and *B. longum* than the CHB group (Fig. 1c).

TAA was used to generate an experimental mouse model of HE^14,15^. Various degrees of pinna reflex, corneal reflex, tail flexion, escape response, righting reflex, and ataxia were observed in mice after intraperitoneal TAA injection (Supplementary Fig. 1a). The following were observed after TAA induction: aggravated liver necrosis; increased serum alanine aminotransferase (ALT), aspartate aminotransferase (AST), and ammonia levels; elevated cerebral water content; and increased expression of brain inflammatory factors (Supplementary Fig. 1b–f). Subsequently, we evaluated the gut microbial alterations in TAA-treated mice using 16 S rRNA sequencing. The principal coordinate analysis based on the Bray–Curtis distance suggested differences in the overall microbial structure between the TAA-treated mice and the negative control (Fig. 1d). The relative abundance of Muribaculaceae, *Ligilactobacillus*, and *Bifidobacterium* decreased in the TAA-treated mice, while that of *Akkermansia* increased (Fig. 1e). Notably, the level of *Bifidobacterium* decreased in faeces from both patients with HE and TAA-treated mice (Fig. 1f), as confirmed via quantitative polymerase chain reaction (qPCR) (Fig. 1g). The metagenomic analysis suggested a decrease in the abundance of two species under the genus *Bifidobacterium* (namely, *B. longum* and *B. pseudocatenulatum*) (Fig. 1h).

### Oral transfer of faecal miRNAs from patients with HE exacerbated the disease

**Fig. 2:**
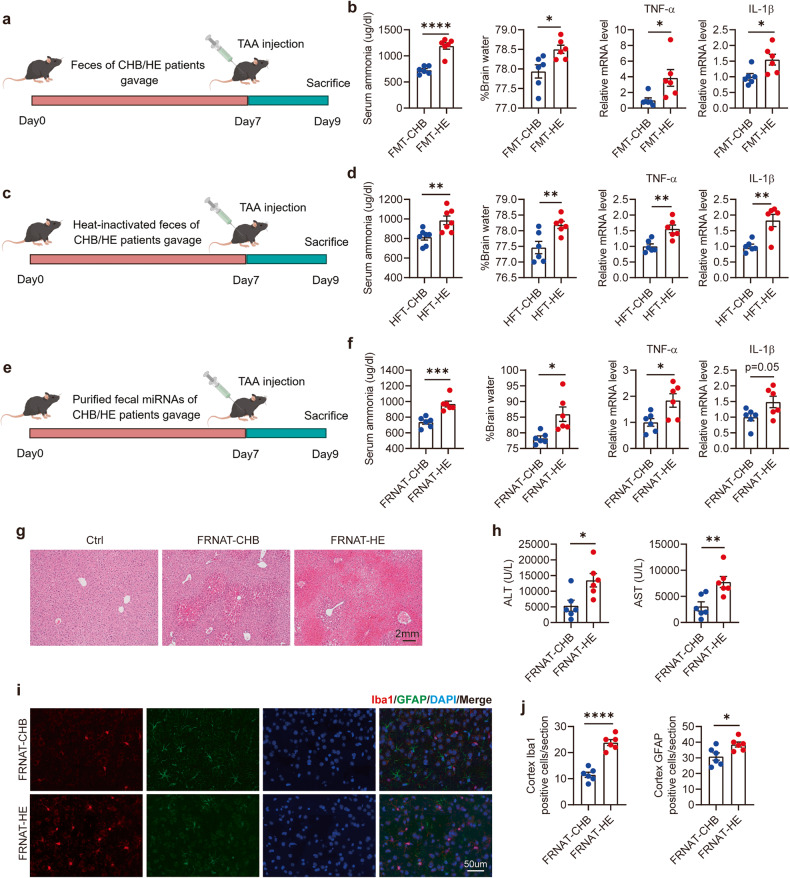
Oral transfer of faecal miRNAs from patients with HE exacerbated the disease. **a**, **b** The effects of faecal transfer on HE. **a** Schematic design. Faeces were collected from patients with HE and CHB, and orally gavage to recipient mice for 7 consecutive days. After that, mice were injected with TAA for inducing HE. **b** Serum ammonia level, brain water content, transcripts of TNF-α, IL-1β in the cerebral cortex. **c**, **d** The effects of the transfer of heat-resident faecal composition on HE. **c** Schematic design. Faeces of patients with HE and CHB were heat-inactivated and orally gavage to recipient mice. **d** Serum ammonia level, brain water content, transcripts of TNF-α, IL-1β in the cerebral cortex. **e**–**j** The effects of faecal miRNAs transfer on HE. **e** Schematic design. Mice were transplanted with small RNA extracted from faeces of patients with HE and CHB. **f** Serum ammonia level, brain water content, transcripts of TNF-α, IL-1β in the cerebral cortex. **g** Histopathological analysis of liver sections; representative pictures of H&E staining are shown. Scale bars, 2 mm. **h** Serum ALT, AST levels. **i** Immunofluorescence staining of Iba1 and GFAP in the cerebral cortex; representative images are shown. Red: Iba1; green: GFAP; blue: DAPI. Scale bars, 50 µm. **j** Statistics form of **i**. *N* = 6 per group. Data are represented as mean ± SEM. *p < 0.05, **p < 0.01, ***p < 0.001, ****p < 0.0001, determined by unpaired Student’s *t*-test (**b**, **d**, **f**, **h**, **j**).

Faecal microbial colonisation from patients with cirrhosis has been reported to result in a higher degree of neuroinflammation in mice, irrespective of cirrhosis, as compared with that from healthy humans^16^. To investigate whether the faecal composition in patients with HE had pathogenic or protective properties, we orally transferred faeces from patients with HE and CHB to recipient mice and subsequently injected TAA for HE induction (Fig. 2a). Considering that inflammation is an important contributor to HE progression, we, therefore, assessed neuroinflammation in these animal models. Furthermore, given that ammonia acts in concert with neuroinflammation to induce cognitive impairment in HE^17^ and that HE is also characterised by cerebral oedema^18,19^, we determined the cerebral water content^20,21^, brain inflammatory factors (tumour necrosis factor-α [TNF-α] and interleukin [IL]-1β)^22,23^, and serum ammonia level to evaluate the disease severity. We found that the transfer of faeces collected from patients with HE resulted in elevated serum ammonia levels, a higher degree of neuroinflammation, and brain oedema in TAA-treated mice compared to those collected from patients with CHB (Fig. 2b). These data indicated that the transfer of faeces from patients with HE aggravated HE compared to those from patients with CHB.

To investigate whether live bacteria in the faeces were responsible for these effects, we heated the inactivated faeces before transfer (Fig. 2c). The heat-inactivated faeces from patients with HE still aggravated the disease compared to those from patients with CHB (Fig. 2d), implying that live microbes were not necessary.

Previous studies confirmed that miRNAs were heat-resistant^24^ and that miRNAs enriched in faeces from diseased persons could modulate bacterial transcripts and growth, resulting in disease amelioration^12,13^. Hence, we subsequently investigated whether faecal miRNAs from patients with HE exerted these effects by purifying the total miRNAs in faeces from patients with HE and CHB and transplanting them into the mice (Fig. 2e). The results showed that the transfer of purified faecal miRNAs from patients with HE significantly increased the serum ammonia levels and cerebral water content and aggravated the neuroinflammation compared to those from patients with CHB (Fig. 2f). Liver pathology revealed widespread necrosis, immune cell infiltration, and haemorrhage (Fig. 2g) and increased serum ALT and AST levels (Fig. 2h).

Microglial activation can promote the production of pro-inflammatory mediators. For the evaluation of neuroinflammation in TAA-treated mice, we detected the expression of ionised calcium-binding adaptor molecule 1 (Iba1) and glial fibrillary acidic protein (GFAP), which are microglial- and astrocyte-specific markers via immunofluorescence staining. Microglial proliferation was significantly increased in recipients of purified faecal miRNAs from patients with HE (Fig. 2i, j). These results indicated that miRNAs in faeces might be crucial to HE progression.

### Identification of differentially expressed faecal miRNA in HE

**Fig. 3:**
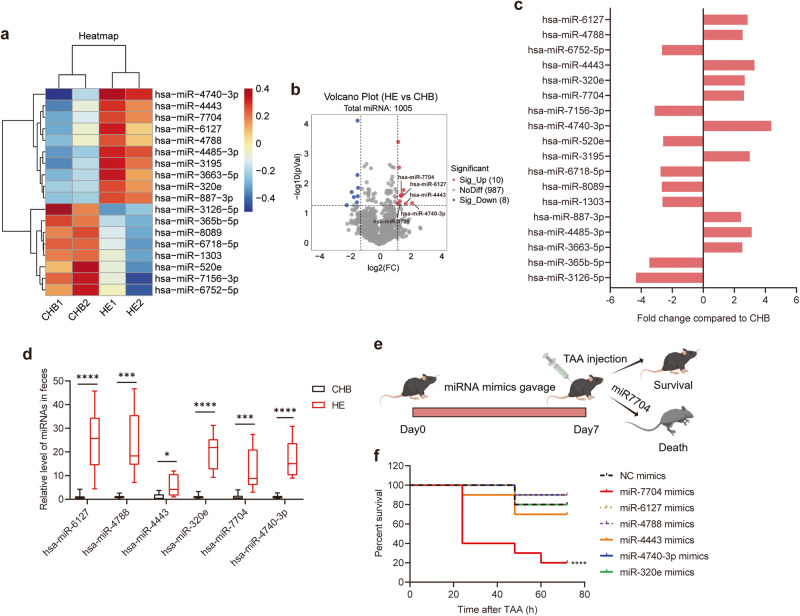
Identification of differentially expressed faecal miRNA in HE. **a**–**c** Microarray analysis of miRNA expression profiles. RNA was isolated from faeces of patients with HE and CHB. **a** Heatmap of miRNA expression (*p* < 0.05, fc > 2.4). **b** Volcano plot analysis of miRNA microarray between CHB and HE groups. **c** Differentially expressed miRNAs were screened by fold change and arranged according to abundance (from top to bottom). **d** Relative abundance of hsa-miR-6127, hsa-miR-4788, hsa-miR-4443, hsa-miR-320e, hsa-miR-7704 and hsa-miR-4740-3p were verified by qPCR, *n* = 10. Data range from min to max, centre lines represented as median, bounds of box represented as first quartile and third quartile. Orally gavage of the above six miRNA to the recipient mice, then injected them with TAA. **e** Schematic design. **f** Survival curve, *n* = 10. Data are represented as mean ± SEM. *p < 0.05, **p < 0.01, ***p < 0.001, ****p < 0.0001, determined by unpaired Student’s *t*-test (**d**) or linear regression analysis (**f**).

As previously reported, faeces from diseased individuals could be enriched with miRNAs, which exerted preventive effects^12,13^. To further identify the faecal miRNAs generated in HE, we conducted a miRNA microarray analysis on faeces collected from patients with HE and CHB. The microarray analysis of miRNA expression profiles revealed that 10 miRNAs had significantly higher abundances in faeces from patients with HE than in faeces from patients with CHB. Meanwhile, the abundance of 8 miRNAs was lower (Fig. 3a, b). Specifically, several significantly different miRNAs were identified based on fold change, *p*-value, and abundance, including hsa-miR-6127, hsa-miR-4788, hsa-miR-4443, hsa-miR-320e, hsa-miR-7704, and hsa-miR-4740-3p, which were elevated in faeces from patients with HE compared to that from patients with CHB (Fig. 3c).

Using qPCR, we also confirmed the increase in these miRNAs in faeces from patients with HE (Figs. 3d and S2a). These results identified differentially expressed faecal miRNAs in HE. To explore whether the miRNAs enriched in faeces from patients with HE could influence disease progression, we synthesised the top 6 most abundant miRNAs. We administered them to mice for 7 consecutive days before TAA injection (Fig. 3e). The miRNA levels in faeces were detected after miRNA administration. The results indicated a significantly increased miRNA content, suggesting the effectiveness of intragastric treatment (Supplementary Fig. 2b). The miR-7704-treated mice exhibited a higher mortality rate than the controls. However, no similar effect was observed in the other miRNA-treated groups (Fig. 3f). Instead of administering a lethal dose of TAA, we used a normal dose to observe survival. Additionally, to assess the severity of HE, we determined the serum ALT, AST, and ammonia levels as well as the cerebral water content in each group, except for the miR-7704-treated group, which also showed no significant difference (Supplementary Fig. 2c).

### Oral administration of hsa-miR-7704 aggravated HE in a microbiome-dependent manner

**Fig. 4:**
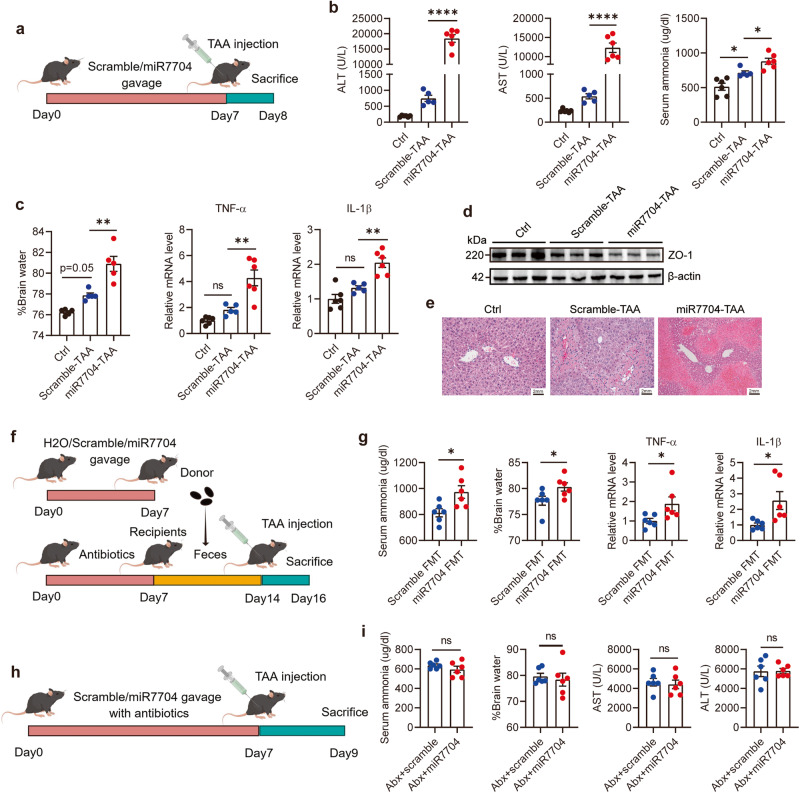
Oral administration of hsa-miR-7704 aggravated HE in a microbiome-dependent manner. **a**–**d** The effects of oral administration of miR-7704 on HE. **a** Schematic design. **b** Serum ALT, AST and ammonia levels. **c** Brain water content and transcripts of TNF-α, IL-1β. **d** Expression of ZO-1 in the cerebral cortex. **e** Histopathological analysis of liver sections; representative pictures of H&E staining are shown. Scale bars, 2 mm. The effects of the transfer of faeces from miR-7704-treated mice on HE. **f** Schematic design of faecal microbiota transplantation. **g** Serum ammonia level, brain water content, transcripts of TNF-α, IL-1β in the cerebral cortex. The effects of miR-7704 on HE in antibiotics treated mice. **h** Schematic design. **i** Serum ALT, AST and ammonia level, brain water content. Data were analysed with one-way ANOVA (**b**, **c**) or unpaired Student’s *t*-test (**g**, **i**). Values are mean ± SEM, *p < 0.05, **p < 0.01, ***p < 0.001, ****p < 0.0001. ns no significance.

We observed that oral administration of miR-7704 resulted in higher mortality in TAA-treated mice. Accordingly, we administered synthetic miR-7704 and scrambled sequence to the recipient mice, injected a lower TAA dose (i.e., 100 mg/kg) into these mice, and sampled them at 24 h after injection to observe pathological changes (Fig. 4a). As expected, the serum ALT, AST, and ammonia levels dramatically increased in miR-7704-pretreated mice (Fig. 4b). Furthermore, severe brain oedema and elevated transcription levels of TNF-α and IL-1β were noted in the cerebrum of miR-7704-pretreated mice (Fig. 4c), indicating neuroinflammation. The expression of zonula occludens-1 (a tight junction protein) (ZO-1) in the cerebral cortex was also reduced in miR-7704-pretreated mice (Fig. 4d), suggesting damage to the BBB. Haematoxylin and eosin staining of liver sections from TAA-treated mice showed evident destruction of the liver architecture, inflammatory cell infiltration, and necrosis, and miR-7704 intensified this type of liver damage (Fig. 4e). These results indicated that oral administration of miR-7704 aggravated HE.

Because hsa-miR-7704 does not have homologous mature sequences in mice, we surmised that it regulates the HE phenotype by disrupting the gut microbiota. To investigate whether oral administration of miR-7704 mimics disordered microbiome, we collected faeces from miR-7704-treated mice on day 7 without TAA injection and then transferred them to the recipient mice pretreated with antibiotics for microbiome depletion (Fig. 4f). The recipients that received faeces from miR-7704-pretreated donor mice exhibited higher serum ammonia levels and a higher degree of neuroinflammation (Fig. 4g). Furthermore, we treated mice with antibiotics and miR-7704 mimics at the same time. We observed that antibiotic treatment abrogated the effect of oral miR-7704 in mice (Fig. 4h–i). These results suggested that hsa-miR-7704 shaped the disordered microbiome, contributing to the development of HE. Thus, we hypothesised that hsa-miR-7704 administration altered specific gut microbes that regulate disease progression.

### hsa-miR-7704 suppressed the growth and adhesion of B. longum via proB

**Fig. 5:**
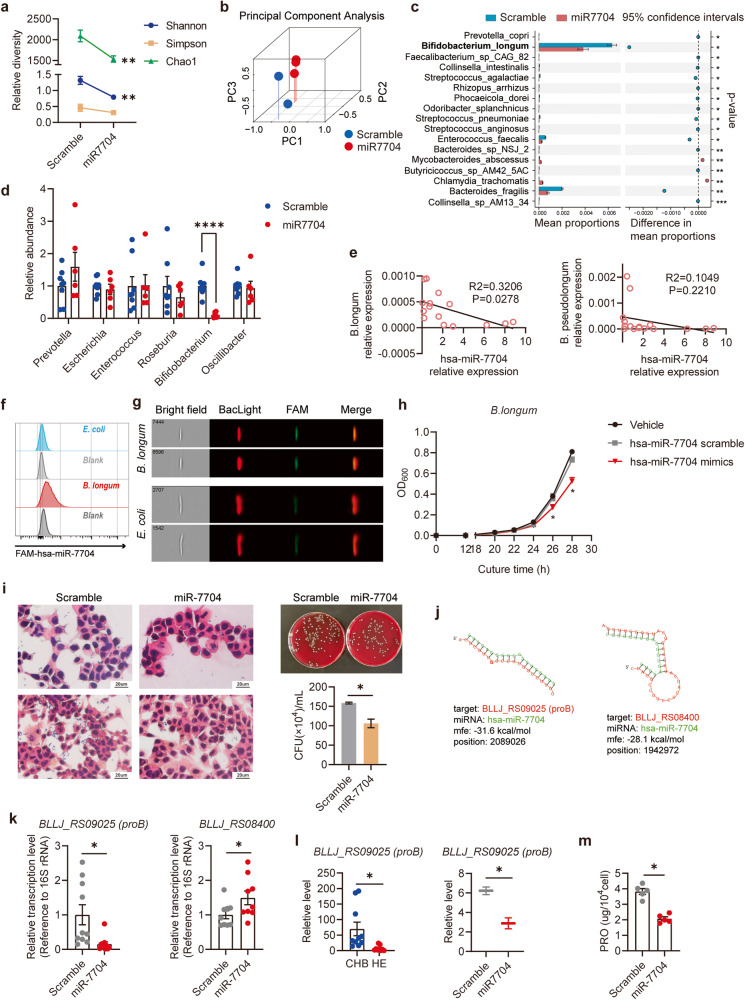
hsa-miR-7704 suppressed the growth and adhesion of *B. longum* via *proB* inhibition. **a** Diversity and **b** PCA of microbiota in faeces of invitro miR7704-treated and control group. **c** STAMP was performed on species level. Top17 species screened by fold change and *P* value were shown. **d** Relative abundance of faecal microbiota, determined by qPCR. **e** Correlation between the relative abundance of *B. longum*/ *B. pseudolongum* (determined by metagenomics analysis) and the relative expression of hsa-miR-7704 (determined by qPCR) in faeces from patients with HE and CHB (*n* = 15). Linear regression was performed. **f**–**i** Bacteria were grown in the presence of hsa-miR-7704 mimics and scramble (2 µM, 4 h). **f** Proportions of FAM-hsa-miR-7704 positive bacteria, detected by flow cytometry. **g** The bacterium (BacLight Red) and miRNA (FAM) were detected and pictured by Imaging streaming. **h** Growth curve of *B. longum*. **i** *B. longum* pretreated with hsa-miR-7704 mimics or scramble were cultured with HT-29 cells (5 × 10^7^ CFU/mL, 4 h), and gram-stained. Representative images of two different densities are shown. Scale bars, 20 µm. Quantification of the adhering bacteria. **j** Schematic diagram of the predicted target sites. **k** Transcripts of the predicted targeting genes, normalized to 16S rRNA. *N* = 10. **l** Relative expression of the predicted targeting gene BLLJ_RS09025 [*proB*] was verified by metagenomics analysis. **m** Proline levels of miR7704-treated faeces and control. Data were analysed with unpaired Student’s *t*-test (**a**, **d**, **i**, **k**, **l**, **m**), linear regression analysis (**e**), or one-way ANOVA (**h**). Values are mean ± SEM, *p < 0.05, **p < 0.01, ***p < 0.001, ****p < 0.0001.

Next, we investigated whether and how miR-7704 affected the gut microbes. The faeces of patients with CHB were collected and co-cultured with hsa-miR-7704 mimics in vitro. The overall microbiome structure of the patients’ faeces changed, and the microbial diversity decreased after miR-7704 treatment according to metagenomic analysis (Fig. 5a-b). Notably, the abundance of *B. longum* in the miR7704-treated group significantly decreased compared to that in the control group (Fig. 5c). Moreover, the relative abundance of *Bifidobacterium* decreased in the faeces of miR-7704-gavaged mice (Fig. 5d). Correlation analysis showed a negative correlation between the abundance of *B. longum* and the expression of hsa-miR-7704 in patients’ faeces. Meanwhile, the abundance of *B. pseudolongum* did not significantly correlate with the expression of hsa-miR-7704 (Fig. 5e). These findings suggested that hsa-miR-7704 might reduce the abundance of *B. longum* in faeces.

The entry of miRNAs into the bacteria has a certain specificity^11^. To determine whether hsa-miR-7704 specifically entered *B. longum*, we used flow cytometry to detect the proportion of bacteria entered by miR-7704 mimics. We found that miR-7704 could enter *B. longum*, but not *E. coli* (Fig. 5f). We also found colocalisation of miR-7704 with *B. longum* (Fig. 5g). These results showed that miR7704 specifically enters *B. longum*.

We cultured *B. longum* with hsa-miR-7704 mimics in an in vitro anaerobic fermentation system to explore whether hsa-miR-7704 influenced the growth of *B. longum*. Notably, we observed that the miR-7704 mimics inhibited the growth of *B. longum* (Fig. 5h). In our previous study, intestinal proteins have been shown to regulate the adhesion of probiotic bacteria to the intestinal epithelial cells^25^. Accordingly, we co-cultured *B. longum* pretreated with miR-7704 mimics or scrambled sequence and HT-29 cells for 4 h and then observed the adhesion by Gram staining. Quantification of adhesive bacteria revealed that HT-29 cells cultured with miR-7704-pretreated *B. longum* had higher amounts of adhesive bacteria than scrambled sequence-pretreated *B. longum* (Fig. 5i). These results showed that miR-7704 suppressed the growth and adhesion of *B. longum*.

Next, we investigated how miR-7704 regulated the growth and adhesion of *B. longum* by blasting the hsa-miR-7704 sequence against the whole genome sequence of *B. longum*. We noted that the potential targets of hsa-miR-7704 were two genes (locus tag BLLJ_RS09025 [*proB*] and locus tag BLLJ_RS08400) (Fig. 5j and S3). The relative expression of *proB* detected by qPCR was significantly lower when *B. longum* was incubated with miR-7704 mimics than with the scrambled sequence. In contrast, the expression of BLLJ_RS08400 was higher (Fig. 5k). We also observed that, according to metagenomic sequencing, the relative level of *proB* decreased in faeces from patients with HE, as well as in faeces of those in the miR7704-treated group (Fig. 5l). The protein product of *proB* is glutamate 5-kinase, which controls the production of proline/ornithine^26^. *B. longum* treated with miR-7704 mimics had significantly lower proline levels than those in the control group (Fig. 5m). This indicated that miR-7704 inhibited the expression of *proB* and proline production in *B. longum*.

To determine the role of *proB* in regulating growth and adhesion, we initially attempted to knock out *proB* in *B. longum* but failed. However, we knocked out *proB* in *E. coli* str. K-12 substr. MG1655 and observed that the modified strain grew slower and took longer to reach a plateau (Supplementary Fig. 4a). We also found that the knockout of *proB* decreased the adhesion ability of the *E. coli* strain (Supplementary Fig. 4b). The protein product of BLLJ_RS08400 is NAD( + )/NADH kinase. We overexpressed BLLJ_RS08400 in *E. coli* and observed no significant difference in cell growth or adhesion (Supplementary Fig. 4c–d). We also cultured *E. coli* with hsa-miR-7704 mimics or scrambled sequences in vitro and observed no significant difference in the growth rate or *proB* expression (Supplementary Fig. 4e-f). These results showed that hsa-miR-7704 inhibited the expression of *proB* in *B. longum*, thereby suppressing growth and adhesion.

### B. longum and its metabolite acetate alleviated HE by inhibiting microglia activation and ammonia production

**Fig. 6:**
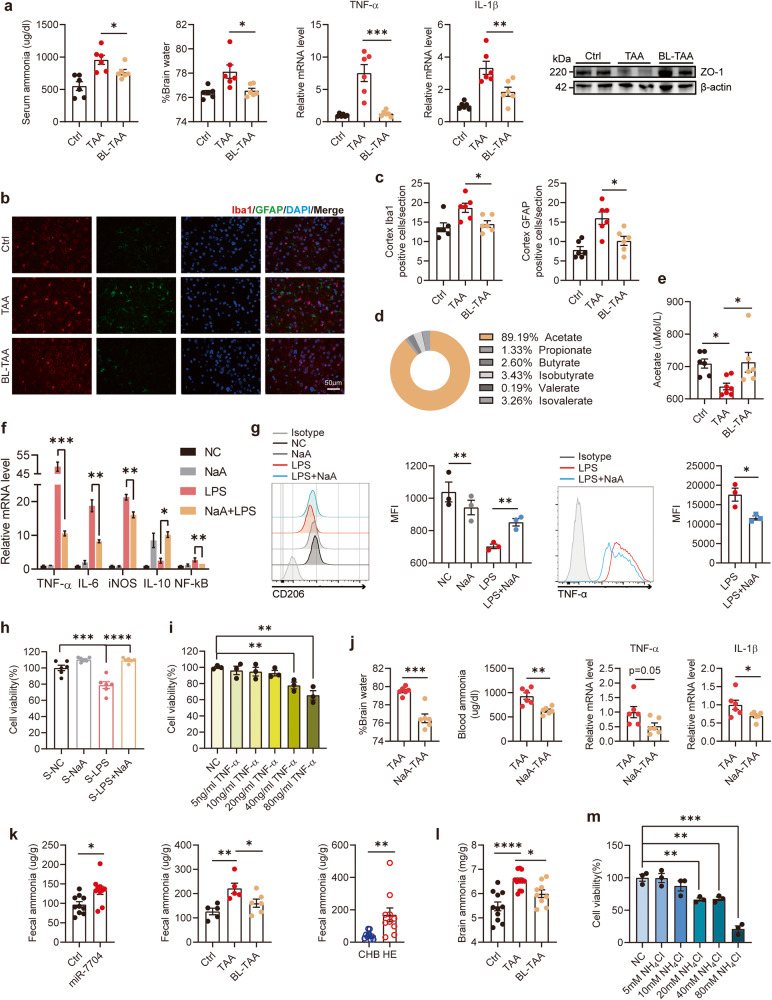
*B. longum* and its metabolite acetate alleviated HE by inhibiting microglia activation and ammonia. **a**–**c** The effects of oral administration of *B. longum* (1 × 10^10^ CFU/mL, 200 µL, 7 days) on TAA-induced mice. **a** Serum ammonia level, brain water content, transcripts of TNF-α, IL-1β, expression of ZO-1 in the cerebral cortex. **b** Immunofluorescence staining of Iba1 and GFAP in the cerebral cortex. Red: Iba1; green: GFAP; blue: DAPI. Scale bars, 50 µm. **c** Statistics form of **b**. **d** Distribution of various SCFAs in the metabolites of *B. longum*, determined by Gas Chromatography-Mass Spectrometer (GC-MS). **e** Quantification of serum acetate level in the different groups of mice. **f**–**h** BV2 cells were cultured with sodium acetate (NaA, 5 mM, 3 h) and LPS (100 ng/mL, 3 h). **f** Relative gene expression of inflammatory cytokines. **g** CD206 expression and TNF-α expression were analysed by flow cytometry. **h** Cell viability of HT-22 cells stimulated by the supernatant collected from BV2 cells for 24 h, determined by cell counting kit-8 (CCK-8). **i** Cell viability of HT-22 cells stimulated by different concentration of TNF-α for 24 h, determined by CCK-8. **j** Serum ammonia level, brain water content, transcripts of TNF-α, IL-1β in the cerebral cortex. **k** Ammonia levels in faeces. **l** Ammonia levels in the brain tissues of mice. **m** Cell viability of HT-22 cells stimulated by different concentration of ammonium chloride (NH_4_Cl) for 24 h. Data were analysed with one-way ANOVA (**a**, **c**, **e**–**h**, **k**) or unpaired Student’s *t*-test (**g**, **i**-**k**, **m**). Values are mean ± SEM, *p < 0.05, **p < 0.01, ***p < 0.001, ****p < 0.0001.

*B. longum* is thought to be a probiotic microbe that promotes intestinal health and confers protection against depression, stress, and ageing through the gut-brain axis^27–29^. We further confirmed the effect of *B. longum*, as oral administration of *B. longum* alleviated TAA-induced HE in mice (Fig. 6a). Microglial and astrocyte activation in the cortex, as assessed by the immunoexpression of Iba1 and GFAP, were significantly lower in the *B. longum*–TAA group than in the TAA group (Fig. 6b, c). Moreover, the immunoexpression of Iba1 and GFAP in the cerebral cortex was significantly higher in the miR-7704–TAA group than in the TAA group, indicating neuroinflammation (Supplementary Fig. 5a, b).

*B. longum* has been reported to produce short-chain fatty acids (SCFAs) with anti-inflammatory effects^28,30^. Using an in vitro anaerobic fermentation system, we confirmed that acetate was the major SCFA produced by *B. longum* (Fig. 6d). We then determined that the plasma level of acetate was higher in the *B. longum*-treated group than in the TAA group (Fig. 6e). We investigated which immune cells were regulated by acetate during HE progression and found that *B. longum* treatment decreased the proportion of F4/80^+^ CD11b^+^ CD11c^+^ macrophages (M1-type) and increased the proportion of F4/80^+^ CD11b^+^ CD206^+^ macrophages (M2-type) in the liver. However, the proportions of other immune cells (CD4^+^/CD8^+^ T cells, NK cells, and NKT-like cells) were not significantly different (Supplementary Fig. 6a–c).

Conversely, the proportion of M1-type macrophages increased, whereas the proportion of M2-type macrophages was lower in the miR-7704–TAA group than in the TAA group (Supplementary Fig. 6d). Liver immune cells were collected from naïve mice and cultured with acetate (NaA). We observed that acetate promoted the differentiation of primary macrophages to M2-type macrophages rather than to M1-type macrophages in vitro (Supplementary Fig. 6e). However, miR-7704 did not directly affect the M1/M2 polarisation of macrophages (Supplementary Fig. 6f), indicating an essential role of the gut microbiota.

The microglia are critical tissue-resident macrophages in the nervous system and are activated in HE^17,31^. We hypothesised that acetate would inhibit microglial activation. The microglia were stimulated using lipopolysaccharide (LPS) to produce the M1 phenotype^31^. We stimulated mouse microglial cells (BV2 cells) with LPS and found that acetate inhibited the expression of M1-type inflammatory factors (TNF-α, IL-6, iNOS) and promoted the expression of M2-type anti-inflammatory factors (IL-10). The activation of NF-κB induced by LPS was also inhibited by acetate (Fig. 6f). Moreover, the expression of CD206, detected via flow cytometry, was decreased by LPS stimulation compared to that in the negative control. This effect was blocked using acetate. Conversely, the production of TNF-α by NaA-cultured BV2 cells was reduced (Fig. 6g).

In addition to their pathological responses, the microglia also regulate neuronal activity^32^. The viability of neuronal cells (HT22 cells) was inhibited by the supernatant collected from LPS-stimulated BV2 cells. The viability of HT22 cells was higher in the supernatant collected from the LPS + NaA group than in the LPS group (Fig. 6h). Therefore, we hypothesised that the cytokines secreted during microglial activation are important for regulating neuronal cells. Because the TNF-α level was markedly changed in both the mouse and in vitro cell models, we investigated whether TNF-α could regulate neuronal cell activity. As expected, we found that the viability of HT22 cells could be inhibited by TNF-α recombinant protein (Fig. 6i). We further confirmed the protective effect of acetate in vivo, as treatment of sodium acetate alleviated TAA-induced HE in mice (Fig. 6j).

Hyperammonaemia is crucial for the development of HE. Ammonia levels are correlated with disease severity and predict mortality. During HE, elevated circulating ammonia levels are caused by increased ammonia production by the gut bacteria and a limited capacity for ammonia detoxification^17,33^. Notably, we found that faecal ammonia levels were elevated in miR-7704-treated mice (Fig. 6k). The faecal ammonia levels in TAA-treated mice were higher than that in the control group but decreased when treated with *B. longum* (Fig. 6k). The changes in ammonia levels in the cerebrum were similar to those in the faecal ammonia levels (Fig. 6l). We also determined that faecal ammonia levels in patients were elevated (Fig. 6k). High ammonia levels inhibit neuronal cell activity^17,34^. We further verified this effect, as HT22 cell viability was inhibited by high concentrations of ammonium chloride (NH_4_Cl) (Fig. 6m). All these results indicated that *B. longum* and its metabolite acetate alleviated HE by inhibiting microglial activation and ammonia production.

## Discussion

HE is widely accepted to be closely associated with the gut microbiota. Factors related to disorders of the gut environment, such as systemic inflammation, ammonia, and endotoxaemia, act as major mechanisms in the pathogenesis of HE^4^. Previous studies have shown that the overall microbial structure in patients with HE is altered^35^. Notably, we compared the faecal microbiota composition between patients with HBV-ACLF with HE and those with CHB. However, other studies mostly compared the composition between patients with cirrhosis with and without HE. We also used TAA to build an experimental mouse model of HE and observed alterations in the gut microbiota. The alterations at different taxonomy levels were generally similar but slightly different from another study^14^. Several bacteria that have been reported to be associated with HE also changed in our model, such as *Akkermansia*, which may be protective or disruptive in liver cirrhosis^36,37^.

The gut microbiome communicates with the brain during the progression of HE. Manipulating the microbiome is an effective approach to treatment. A previous study reported that faecal microbial colonisation from patients with cirrhosis led to a higher degree of neuroinflammation in mice, irrespective of cirrhosis, compared to healthy humans^16^. In line with this, our data showed that the transfer of faeces from patients resulted in a higher degree of neuroinflammation in mice than in patients with CHB. Furthermore, heat-inactivated faeces and faecal miRNAs had similar effects. This may imply that faecal compositions play a regulatory role during the progression of HE, where live microbes are not necessary.

Circulating miRNAs are associated with the progression and prognosis of cirrhosis and ACLF^38^. miRNAs mediate communication between the liver and brain^39^. In mice with ALF-induced HE, miRNA expression is altered in the cerebral cortex^40^. Moreover, plasma extracellular vesicles (EV) can play a role in inducing immune changes in minimal HE^41^. We have previously studied the role of miRNA in HBV-related hepatocellular carcinoma^42^. Nevertheless, the involvement of gut miRNAs in HE progression has not yet been reported. Here, we found that hsa-miR-7704 enriched in faeces from patients with HE-aggravated disease in a microbiome-dependent manner. We verified the association between gut miRNA and HE progression for the first time.

Gut miRNAs can enter microbes and subsequently influence bacterial growth and gene expression^11–13,43,44^. In line with this, we found that hsa-miR-7704 inhibited the growth and adhesion of gut commensal *B. longum* and suppressed the gene expression of *proB* in *B. longum*. The function of *proB* in *Bifidobacterium* is unreported and is unstudied in the microbiome. This gene encodes glutamate 5-kinase (G5K) and catalyses the rate-limiting first step of proline synthesis (and ornithine synthesis in mammals). It is a key regulatory point of these routes since it is the subject of feedback allosteric inhibition by proline or ornithine^45,46^. Proline is an important osmotic regulator and a free radical scavenger. As expected, the proline level significantly decreased in *B. longum* after treatment with miR-7704. Our attempt to directly verify the function of this gene in *B. longum* failed owing to the lack of a mature knockout system and the harsh growth conditions of gram-positive anaerobes in this bacterium.

In addition, the knockout of this gene may affect bacterial growth and metabolism, which may also be an important reason for the difficulty in knockout. A previous study used *E. coli* as a vector to verify the function of a gene in *Akkermansia muciniphila*^12^. Thus, we showed that the knockout of *proB* inhibited the growth of *E. coli*. Although miR-7704 cannot enter *E. coli* and, therefore, cannot affect it, the experiment aids in understanding the role of the *proB* gene in bacteria. Our results and previous studies have also shown that miRNAs can selectively enter bacteria^11^. It is noteworthy that miRNAs may shape the gut microbiome by anchoring specific genes and microbes.

There is an interaction in the microbiome. Changes in the abundance of one type of bacteria can often cause changes in others. As a small RNA molecule, miR-7704 may have several targets. However, miR-7704 selectively enters the bacteria; it only regulates specific bacteria. The internal interactions of the microbiome affect the abundance of non-target bacteria. Therefore, it is difficult to identify all the target bacteria of miR-7704 by analytical means. Further research is needed to determine other bacteria regulated by miR-7704 and whether these bacteria play a role in disease. Here, we focus on the regulatory effects of miR-7704 on *B. longum*, which is crucial in the HE process.

Our previous reports have investigated the role of gut microbiota and immune cells in hepatic and intestinal diseases^25,47–49^. *B. longum* is one of the most common probiotics that can inhibit the growth of harmful bacteria, synthesise antibiotics needed by the human body, and stimulate the human immune system to improve the body’s resistance to disease^50–52^. Probiotic therapies, including those using *B. longum*, have beneficial effects on HE^53,54^. SCFAs serve as a link between host nutrition and intestinal homeostasis and have been reported to have important immunomodulatory functions. Consistent with these studies, we showed that *B. longum* and its metabolite acetate, a type of SCFA, alleviated HE by inhibiting ammonia, microglial activation, and M1-type macrophage polarisation.

Systemic inflammation and ammonia toxicity have been described to be associated with brain oedema, which leads to high mortality. Alterations in the gut-liver-brain axis present an inflammatory state, including reduced BBB permeability, microglial activation, and production of pro-inflammatory factors, which can lead to neuronal death^55^. Ammonia is primarily produced by the gut microbiota. Weakened BBB and increased circulating ammonia levels can cause toxic effects on the brain, which is the most widely accepted hypothesis of the development of HE. Hyperammonaemia causes microglial activation in both acute and chronic HE^56^. In this study, we highlighted the role of inflammatory states in HE instead of neuropsychiatric abnormalities. We measured circulating ammonia levels to evaluate ammonia toxicity and cerebral TNF-α and IL-1β expression to evaluate neuroinflammation. The brain water content was also measured to assess the degree of brain oedema. This may be more feasible for observation than for psychoneurological testing.

However, there were a few limitations in our research. The sample size of the patients needs to be increased. The research on the overall changes of gut microbiota and miRNA during the progression of HE needs to be further enriched. Our assessment of neuropsychiatric performance in mice was inadequate due to device limitations. Orally delivered synthetic miRNAs survive the digestive system as vesicles or non-vesicles^11–13,43^. In line with this, we showed that synthetic miR-7704 can survive digestion and modulate gut microbiota. However, the mechanisms by which oral miRNA maintains integrity in the intestine remain to be explored. Because hsa-miR-7704 does not have homologous sequences in mice, we could not use a miRNA antagonist in a mouse model to evaluate the feasibility of treatment, which has some drawbacks in application and treatment.

**Fig. 7:**
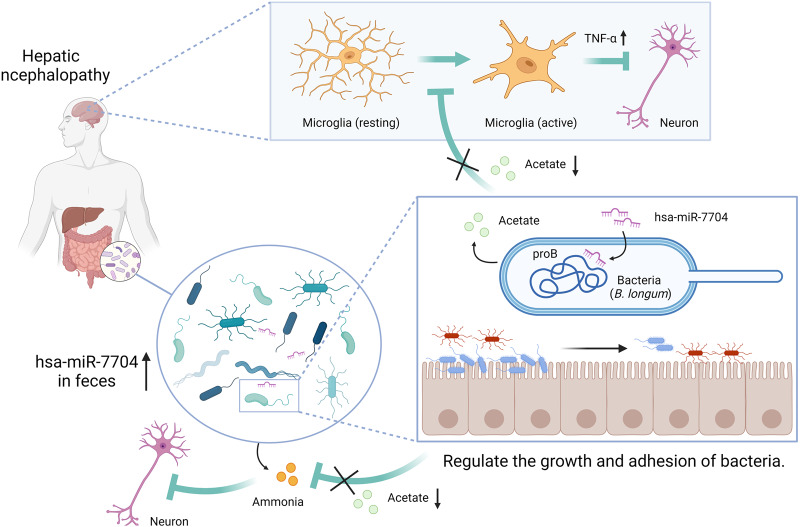
Molecular model depicting the mechanism of hsa-miR-7704 exacerbation of HE through inhibition of *B. longum*. Faecal hsa-miR-7704 enriched in patients with HE inhibits the growth and adhesion of *B. longum* by suppressing *proB*. Reduction of acetate results in overactivation of microglia and hyperammonaemia, thus inhibiting neuronal activity and aggravating HE.

In conclusion, in the present study, we identified a miRNA enriched in patients’ faeces that could specifically modulate the microbiome, further influencing the pathogenesis of HE (Fig. 7). We explained the mechanisms underlying the regulation of specific microbes by miRNAs. Our findings provide insights into the gut miRNAs that shape the gut microbiome and may exhibit therapeutic potential.

## Methods

### Human fecal specimens

Human fecal specimens were collected from 10 CHB subjects (8 males, average 51 years of age) and 9 HE subjects (7 males, average 54 years of age). A protocol approved by the Clinical Research Ethics Committee of the First Affiliated Hospital, Zhejiang University School of Medicine, was followed by all subjects (NO. 2021-029). All subjects gave informed consent prior to participating in the studies. All subjects were excluded for cancers, diabetes, alcoholic liver diseases or COVID-19. A patient who received antibiotics within the last two months and during the sampling period was excluded from the study. In order to collect stool samples, Fisher Scientific’s Commode specimen collection system was used, and the samples were stored at −80 °C until further processing was carried out.

### Mice experiments

6-10 weeks old male C57BL/6J mice were purchased from the Shanghai SLAC Laboratory Animal Co., Ltd; All the above mice were kept under specific pathogen-free conditions and the study was approved by the Research Ethics Committee of the First Affiliated Hospital, College of Medicine, Zhejiang University (NO. 2021-07). All experiments complied with the manual of the care and use of laboratory animals published by the US National Institutes of Health. At the experimental endpoints, all experimental mice were anesthetized with 3-5% isoflurane using an inhalation anaesthesia machine, then euthanized with cervical dislocation.

HE was established using TAA (meilunbio, China) intraperitoneal injection similar as described in previous studies with slightly modification^14,15^. Briefly, mice were given a single intraperitoneal (i.p.) injection of 200 mg/kg TAA and were sacrificed at 48 h-post-injection, unless otherwise indicated.

For observing the effect of miR-7704 gavage, the mice were given a lower dose of 100 mg/kg TAA injection and sacrificed at 24 h-post-injection. Blood, liver, brain tissue, and caecal contents were collected for the subsequent test.

For transplantation of human microbiota, 5 mg per subjects of faeces from patients was washed with sterile PBS, centrifuged at 80 *g* for 10 minutes to remove residuals of faeces and suspended in 200 μL PBS, then gavaged daily orally to recipient mice for 7 consecutive days^12^. The inactivation of faeces has been achieved by heating at 80 °C for 60 min in order to kill bacteria while preserving miRNA^24^. For transfer of faecal miRNAs, 10 μg per subjects of miRNA from faeces were isolated using a miRNA isolation kit (Vazyme, #RC201, China), eluted in 200 μL nuclease-free water, and orally administrated once daily for 7 days to recipient mice before injection with TAA.

Treatment with miRNA mimics or scrambled sequence (40 nM, 200 μL daily) was applied by oral gavage dissolved in nuclease-free water every day for 7 consecutive days. We administered mice intragastrically a cocktail of ampicillin (1 g/L, Sigma, US), neomycin trisulfate (1 g/L, Sigma, US), metronidazole (1 g/L, Sigma, US), and vancomycin (500 mg/L, Sigma, US) for 7 days to clear bacteria. For mouse faecal transplantation, a suspension of 5 mg of faeces from donor mice, dissolved in 200 ml of sterile PBS, was presented once daily to recipient mice by orally gavage for 7 days.

For observing the effects of acetate, mice were pretreated with sodium acetate (i.v. 500 mg/kg, Sangon Biotech, China), then injected with TAA 2 hours later. After 24 hours, the mice were given another injection of sodium acetate (i.p. 500 mg/kg). Mice were sacrificed at 48 h-post-TAA-injection.

### Metagenomic sequencing

Metagenomic analysis was carried out at Metware Biotechnologies Co., Ltd. For the preparation of DNA samples, 1 g of DNA was used as input. Using NEBNext® Ultra™ DNA Library Prep Kit for Illumina (NEB, USA), sequencing libraries were generated following the manufacturer’s recommendations, and index codes were added to each sample so that sequences could be associated with it. A sonicated DNA sample was fragmented to 350 bp, and the fragments were end-polished, A-tailed, and ligated with a full-length adaptor so that PCR amplification could be performed to sequence the fragments. After PCR products were purified (AMPure XP system), the libraries were analysed by Agilent2100 Bioanalyzer for size distribution and quantified with real-time PCR. Index-coded samples were clustered using the cBot Cluster Generation System. On an Illumina NovaSeq platform, paired-end reads were generated after cluster generation.

### 16S sequencing analysis

16S sequencing analysis was conducted by Lc-Bio Technologies Co., Ltd. DNA from different samples was extracted using the CTAB according to manufacturer ’s instructions. PCR amplification was performed, and the PCR products were purified by AMPure XT beads (Beckman Coulter Genomics, Danvers, MA, USA) and quantified by Qubit (Invitrogen, USA). The libraries were sequenced on NovaSeq PE250 platform. Alpha diversity and beta diversity were analysed using QIIME2, and pictures were created using R package (v3.5.2).

### Biochemical assays

Blood samples were centrifuged at 3000 g for 10 minutes at room temperature to separate serum. ALT, AST and ammonia concentrations were evaluated with a dry chemistry analyser (FUJI DRICHEM 7000 V, FUJIFILM, Tokyo, Japan). Fecal samples (100 mg) were suspended in 1 ml PBS, and supernatants were obtained by centrifuging at 13000 *g* for 10 minutes. The cerebrum of mice was fully ground in 500ul PBS, and the supernatants were spun at 13000 g for 10 minutes at room temperature to obtain the supernatants. The fecal and brain ammonia levels were also detected by the dry chemistry analyser. The acetate levels were determined with Acetate Colorimetric Assay Kit (Sigma-Aldrich, US). Proline levels were detected using the Proline (PRO) Content Assay Kit (Solarbio, China).

### Histopathology

Mice were sacrificed and their livers and cerebrums were immediately fixed in 10% formalin for 24 hours. Afterwards, the tissue was rinsed with 70% ethanol, graded dehydrated, and embedded in paraffin. H&E staining was performed to estimate liver damage.

### Immunofluorescence staining

Antigen retrieval was performed on paraffin sections of brain tissues by dewaxing, dehydrating with graded alcohol. 10% BSA at room temperature for 30 min was used for blocking. Samples were incubated with primary antibodies (anti-mouse GFAP, anti-mouse Iba1; Abcam) overnight at 4 °C, washed 3 times with PBS, and incubated with secondary antibody for 30 min at room temperature. Finally, DAPI staining solution was added for 10 min and washed 3 times with PBS. Stained sections were viewed with microscope.

### Brain water

The whole brain was immediately removed after death, and half of the brain was dissected. We used a previously described methods to determine brain water content^20,21,57^. The half of the brain was dried at 80 °C for 3 days. Brain water content was then calculated as percentage of water = (1- dry wt/ wet wt) × 100%.

### Quantitative RT-PCR

Total RNA was extracted from tissues and cells using TRIzol reagent (Invitrogen, US) following manufacturer’s instruction. Total bacterial RNA was extracted using an RNAprep pure Cell/Bacteria Kit (TIANGEN, China). HiScript II 1st Strand cDNA Synthesis Kit (Vazyme, China) was used to synthetic cDNA. SYBR Premix Ex Taq kit ChamQ Universal SYBR qPCR Master Mix (Vazyme, China) was used to determine the relative gene expression. Bacterial DNA of feces was isolated using QIAamp DNA Stool Mini Kit (QIAGEN, US). The relative abundance was normalised to the 16S rRNA. The primers were listed in Supplementary Tables 1 and 2.

### Western blotting

The brain sections were cleaved using RIPA lysis buffer with protease inhibitors and phosphorylation inhibitors added. The extracted protein was separated by SDS-PAGE, then transferred onto PVDF membranes. After blocked at room temperature, the samples were cropped and incubated with primary antibody separately (anti-mouse ZO-1; anti-mouse β-actin; Abcam) overnight at 4 °C, washed 3 times, and incubated with HRP-conjugated secondary antibodies (HUABIO, China) for 1 h at room temperature. Finally, Western ECL substrate was used for exposure (Supplementary Fig. 7). All blots or gels derive from the same experiment and that they were processed in parallel.

### miRNA microarrays

Human fecal specimens were analysed with microarray sequence by KangChen Biotech (Shanghai, China). Sample labeling and array hybridisation were performed with Agilent One-Color Microarray-Based Gene Expression Analysis and miRNA Microarray System with miRNA Complete Labeling and Hyb Kit (Agilent Technologies, Palo Alto CA, USA). After that, the hybridisation solution was assembled and scanned with Agilent DNA Microarray Scanner. Finally, we used Agilent Feature Extraction Software to collect and analyse the array images.

### miRNA isolation, measurement, and treatment

Total miRNA was isolated from fecal specimens using a miRNA isolation kit (Vazyme, China). Isolated miRNA was used for cDNA synthesis by using a miRNA 1st Strand cDNA Synthesis Kit (by stem-loop, Vazyme, China). qPCR was performed to quantify miRNA cDNAs using miRNA Universal SYBR qPCR Master Mix (Vazyme, China). Primers are listed in Supplementary Table 3.

miRNA mimics and scramble were synthesised by Sangon Biotech (Shanghai, China); their sequences are listed in Supplementary Table 4. For the mouse experiment, miRNA mimics or scrambled sequence (40 nM, 200 μL daily) was applied by oral gavage dissolved in nuclease-free water every day for 7 days prior to TAA injection.

### miRNA target prediction

The sequence of hsa-miR-7704 (5′-CGGGGUCGGCGGCGACGUG-3′) was blasted against the whole genome sequence of *B. longum* (JCM 1217) using the NCBI blast tool. The matched gene sequences were screened with “Score” and “E values”. The predicted target genes are showed in alignment view. The minimum free energy of secondary structure binding between hsa-miR-7704 and potential targeting *B. longum* RNA was determined and graphed by RNAhybrid^58,59^.

### Bacteria strains, growth, and administration

*B. longum* (JCM 1217) was grown anaerobically at 37 °C in MRS broth (Oxoid). *E. coli* (str. K-12 substr. MG1655) was grown at 37 °C in LB broth (Sangon Biotech). For mice treatment, 1 × 10^9^ freshly cultured bacteria at logarithmic phase were given by oral gavage daily for 7 consecutive days. For in vitro bacteria growth measurements, bacteria were cultured with miRNA mimics and scramble miRNAs in culture medium and monitored as absorbance at 600 nm (OD600). For investigating whether miR7704 enters bacteria. *B. longum* and *E. coli* was cultured in the presence of 2 µM FAM-labeled hsa-miR-7704 mimics for 4 hours, terminated and washed with cold PBS, fixed and detected by flow cytometry.

### Bacterial adhesion assay

HT-29 cells (ATCC number: HTB-38) were cultured in McCoy’s medium for 14 hours. Bacteria at logarithmic phase were harvested and count on Columbia blood agar plates. In experiments investigating the effect of miR-7704 on *B. longum*, bacteria were pretreated by miRNA mimics or scramble sequence. The HT-29 cells were co-cultured with 5 × 10^7^ CFU/mL bacteria for 4 h, and then washed 2 times with sterile PBS. Fixed cells with adhering bacteria were gram-stained and observed by microscope. The HT-29 cells with adhering bacteria were also harvested and suspended in sterile PBS. CFU were count on Columbia blood agar plates for quantification of adhesion ability.

### Bacterial gene knockout

Homologous recombination was used to knockout the *proB* gene in *E. coli* (str. K-12 substr. MG1655). The 50 bp sequences on both sides of *proB* gene to be knocked down were selected as the upper and downstream homologous recombination arms of gene targeting. The upper and downstream homologous recombination arms were designed to be on both sides of the specific primers for kanamycin (Kn) resistance genes, and the long primers for PCR amplification were obtained by chemical synthesis. Then, plasmid pKD4 was used as template, the target fragment of *proB* was amplified by high-fidelity PCR with the above long primers. The electrically transformed competent cells of MG1655 strain were prepared. The pKD46 helper plasmid of Lambda Red recombinant system was transformed into MG1655 competent cells to obtain MG1655/pKD46 cells. Next, the electro-transformed competent cells of MG1655/pKD46 were prepared, and the target fragment of *proB* was directly transformed into these competent cells. The clones carrying Kn resistance genes were cultured at 37°C on the Kn plate, and the temperature-sensitive pKD46 helper plasmid was eliminated. Finally, the positive clones whose *proB* gene was replaced by Kn resistance genes were screened by PCR, which were called MG1655/ΔproB::Kn. Primers are listed in Supplementary Table 5.

### Flow cytometry and image stream

For surface staining, the cell suspension was incubated with fluorescently labeled antibody at room temperature for 20 minutes. For CD206 staining, the samples were fixed and permeabilised with BD Cytofix/Cytoperm Fixation/Permeabilization Solution kit (BD Biosciences, US), and then incubated with fluorescently labeled antibody at 4 °C for 35 minutes. For intracellular TNF-α staining, the samples were stimulated by LPS (100 ng/ml, Beyotime) for 4 h, fixed and permeabilised, and then incubated with fluorescently labeled antibody at 4 °C for 35 minutes. Bacteria were stained using the BacLight™ Red kit (Invitrogen, B-35001). miRNA was labelled with fluorescein amidites (FAM). The monoclonal antibodies used were as follows: APC-Cy7-anti-Mouse F4/80, Pacific Blue-anti-Mouse CD11b, FITC-anti-Mouse CD11c, APC-anti-Mouse CD206, APC-Cy7-anti-Mouse TCRβ, APC-anti-Mouse NK1.1, PerCP-Cy5-5-anti-Mouse CD45, PE-Cy7-anti-Mouse CD3, PE-anti-Mouse CD4, FITC-anti-Mouse CD8, APC-anti-Mouse TNF-α, all purchased from Biolegend; FITC-anti-Human CD86 (20 μl/ test), APC-anti-Human CD206 (20 μl/test), purchased from BD Pharmingen. Flow cytometry or Image Stream was conducted using a BD FACS CantoII or Millipore ISX with fluorochrome-conjugated cells, and the data was analysed using FlowJo software version 10.4 or IDEAS version 6.0.

### Cell culture and treatment

Immortalised cell lines BV2, HT-22, HT-29, was cultured in proper medium supplemented with 10% fetal bovine serum and 1% penicillin-streptomycin solution at 37 °C with 5% CO2. In experiments of investigating the effect of acetate on microglia, cells were challenged with LPS (100 ng/ml, Beyotime) with or without acetate (5 mM, Sangon Biotech) for 3 hours. For measuring the effect of TNF-α and ammonia on neuronal cell activity, cells were cultured with various concentration of ammonium chloride (Sangon Biotech) or TNF-α recombinant protein (MCE) for 24 h.

### Graphical works

Figures 2a, c, e, 3e, 4a, f, h, were created by Figdraw. Figure 7 was created by BioRender.

### Statistical analysis

All data are analysed with GraphPad Prism and presented as mean ± SEM. Unpaired Student’s *t*-test, one-way ANOVA, and linear regression analysis were used to determine significant differences. *p* < 0.05 was considered significant. *p < 0.05, **p < 0.01, ***p < 0.001, ****p < 0.0001, ns represents not significant.

### Supplementary information

Supplementary table and figure

Reporting-summary

## Data Availability

The metagenomic sequencing data, 16S rRNA data, and miRNA microarray data used in this study are openly available in NCBI (PRJNA953603) and GEO (GSE228731; GSE228827).
