# Supplementary material for: Faecal hsa-miR-7704 inhibits the growth and adhesion of Bifidobacterium longum by suppressing ProB and aggravates hepatic encephalopathy
Source: NPJ Biofilms Microbiomes. 2024 Feb 24;10:13. doi: 10.1038/s41522-024-00487-8 (PMC10891095; doi:10.1038/s41522-024-00487-8)
Supplement: Supplementary file 1 — Supplementary table and figure [file 41522_2024_487_MOESM1_ESM.pdf]

Supplementary Table 1 Primers for quantification of bacteria and bacterial gene transcript by qPCR

Gene	5’-3’ Primer sequence
16S	F: ACTCCTACGGGAGGCAGCAGT<br>R: GTATTACCGCGGCTGCTGGCAC
Bifidobacterium	F: CTCCTGGAAACGGGTGG<br>R: GGTGTTCTTCCCGATATCTACA
Escherichia	F: AATTCTAATACGACTCACTATAGGGAGAAGGC<br>TGGACGGCGACKATCCGGTCTTCA<br>R: AAATCCACATTTCTGACGAGG
Enterococcus	F: GCAATTACTGGTGGAGGACCTGG<br>R: TCCAATTCCTTTTGCAAGACCTGC
Prevotella	F: GGTTCTGAGAGGAAGGTCCCC<br>R: TCCTGCACGCTACTTGGCTG
Roseburia	F: GCACTTTAATTGATTTCTTCGG<br>R: TCTTAGTCAGGTACCGTCATTC
Oscillibacter	F: GGCAATGGACGCAAGTCTGACC<br>R: ATTCGTCAGGTACCGTCTTCTRCTC
BLLJ_RS09025 [proB]	F: TGCGTTCGGTCGATTCGGTATTC<br>R: TGCGTTCCACATTGCGGTACTG
BLLJ_RS08400	F: CCGTCATCGTCATCGCTCTG<br>R: CTGCGGTTTGGACGGATTCA

Supplementary Table 2 Primers for quantification of gene expression by qPCR

Gene	5'-3' Primer sequence
TNF-α	F: CAGGCGGTGCCTATGTCTC
	R: CGATCACCCCGAAGTTCAGTAG
IL-1β	F: GAAATGCCACCTTTTGACAGTG
	R: TGGATGCTCTCATCAGGACAG
IL-6	F: CTGCAAGAGACTTCCATCCAG
	R: AGTGGTATAGACAGGTCTGTTGG
iNOS	F: GTTCTCAGCCCAACAATACAAGA
	R: GTGGACGGGTCGATGTCAC
IL-10	F: CTTACTGACTGGCATGAGGATCA
	R: GCAGCTCTAGGAGCATGTGG
GAPDH	F: AGGTCGGTGTGAACGGATTTG
	R: GGGGTCGTTGATGGCAACA

Supplementary Table 3 Primers for quantification of miRNAs expression by qPCR

Gene	5'-3' Primer sequence
hsa-miR-4740-3p	F: GAACAGAAGCCCGAGAGGATCC<br>R: ATCCAGTGCAGGGTCCGAGG<br>RT:GTCGTATCCAGTGCAGGGTCCGAGGTATTCGCACTGGATACGA CGCAGGG
hsa-miR-4443	F: CGCCGTTGGAGGCGTG<br>R: ATCCAGTGCAGGGTCCGAGG<br>RT:GTCGTATCCAGTGCAGGGTCCGAGGTATTCGCACTGGATACGA CAAAACC
hsa-miR-6127	F: AAGAGCGTTGAGGGAGTGGGT<br>R: ATCCAGTGCAGGGTCCGAGG<br>RT:GTCGTATCCAGTGCAGGGTCCGAGGTATTCGCACTGGATACGA CCCTCCC
hsa-miR-320e	F: AAGAGCGTTGAGGGAGTGGGT<br>R: ATCCAGTGCAGGGTCCGAGG<br>RT:GTCGTATCCAGTGCAGGGTCCGAGGTATTCGCACTGGATACGA CCCTCCC
hsa-miR-4788	F: AACACGTGTTACGGACCAGCTAA<br>R: ATCCAGTGCAGGGTCCGAGG<br>RT:GTCGTATCCAGTGCAGGGTCCGAGGTATTCGCACTGGATACGA CGCCTCC

hsa-miR-7704	F: CGGGGTCGGCGGC<br>R: ATCCAGTGCAGGGTCCGAGG<br>RT:GTCGTATCCAGTGCAGGGTCCGAGGTATTCGCACTGGATACGA<br>CCACGTCG
U6	F: CTCGCTTCGGCAGCACA<br>R: AACGCTTCACGAATTTGCGT<br>RT: AACGCTTCACGAATTTGCGT

Supplementary Table 4 Sequences of miRNA mimics and scramble

Gene	5'-3' sequence
mimics miRNA-7704	F: CGGGGUCGGCGGCGACGUG<br>R: CGUCGCCGCCGACCCCGUU
Scramble miRNA-7704	F: CGGGUAGCGAGGCGUCGUG<br>R: CACGACGCCUCGCUACCCG
mimics miRNA-6127	F: UGAGGGAGUGGGUGGGAGG<br>R: UCCCACCCACUCCCUCAUU
mimics miRNA-4788	F: UUACGGACCAGCUAAGGGAGGC<br>R: CUCCCUUAGCUGGUCCGUAAUU
mimics miRNA-4443	F: UUGGAGGCGUGGGUUUU<br>R: AACCCACGCCUCCAAUU
mimics miRNA-320e	F: AAAGCUGGGUUGAGAAGG<br>R: UUCUCAACCCAGCUUUUU
mimics miRNA-4740-3p	F: GCCCGAGAGGATCCGTCCCTGC<br>R: AGGGACGGAUCCUCUCGGGCUU

Supplementary Table 5 Primers for the experiment of *proB* knockout

Gene	5'-3' sequence
proB	F:ATATCATTTTTCCTAAAATTGAATGGCAGAGAATCATG AGTGACAGCCAGggaataggaacttcaagatcccctc
	R:TTCCAGCATCAGCCTGCTCCTTAACGGGTAATCATGTC ATCACGGTGAACtcagaagaactcgtcaagaaggc
proB-out	F: CAACTGCCGCTAGGCTTGCTGATC
	R: GGATGATTTCGCTTTGTGCTTCCAGTTCATC
Kn	F: GTATCCATCATGGCTGATGCAATGC
	R: GCATTGCATCAGCCATGATGGATAC

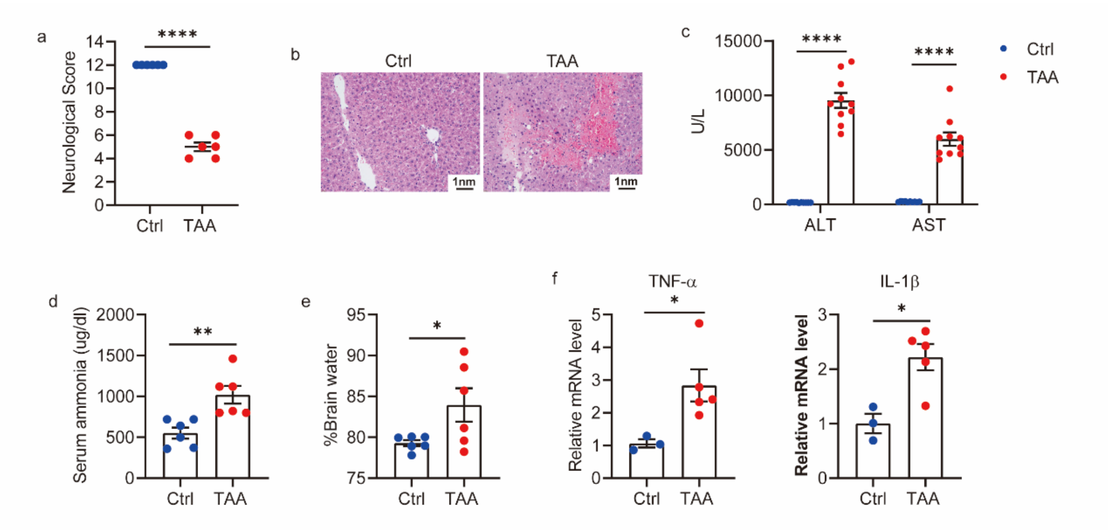

Supplementary Figure 1. Thioacetamide (TAA) induces hepatic encephalopathy in mice. C57/BL6 mice were injected with a single dose of 200mg/kg TAA and sampled at 48 hours-post-injection. (a) Neurological score. (b) Histopathological analysis of liver sections; representative pictures of H&E staining are shown. Scale bars, 1mm. (c) Serum ALT, AST levels. (d) Serum ammonia levels. (e) Brain water content. (f) Transcripts of TNF-α, IL-1β in the cerebral cortex. Data are represented as mean ± SEM and determined by two-way ANOVA or two-tailed Student's t-test. *p < 0.05.

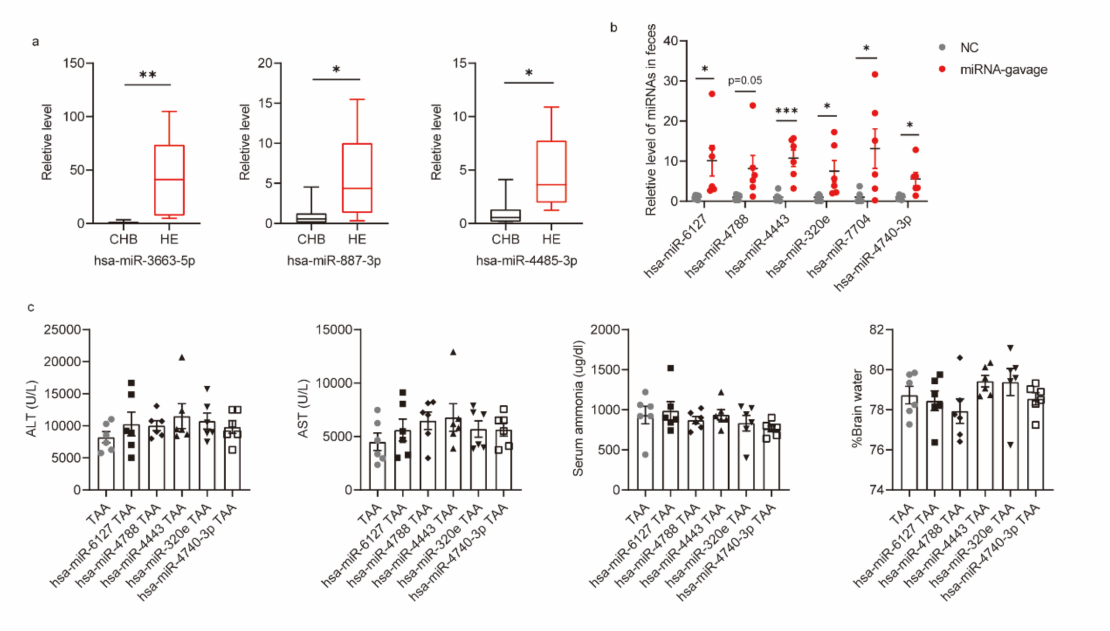

Supplementary Figure 2. Identification of regulatory fecal miRNA during HE. (a) Relative level of hsa-miR-3663-5p, hsa-miR-887-3p, hsa-miR-4485-3p were verified by qPCR. N=10. (b) Relative abundance of miRNA in the feces of mice given gavage. N=6. (c) Serum levels of ALT, AST and ammonia and brain water content in the mice given miRNA gavage. N=6. Error bars denote mean ± SEM; two-tailed Student's t-test was performed, *p < 0.05.

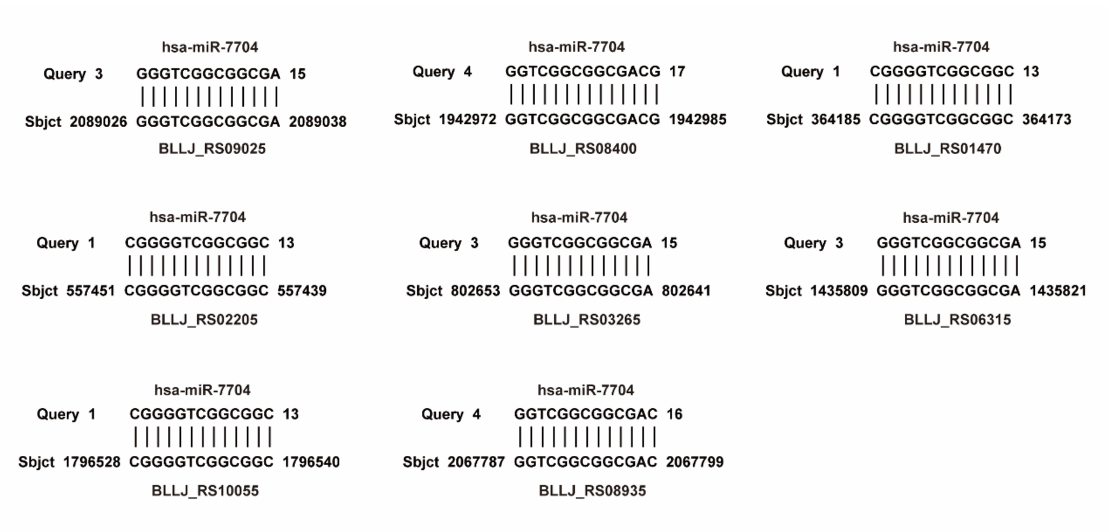

Supplementary Figure 3. Schematic diagram of the putative binding sites of hsa-miR-7704 in the complete sequence of *B. longum*.

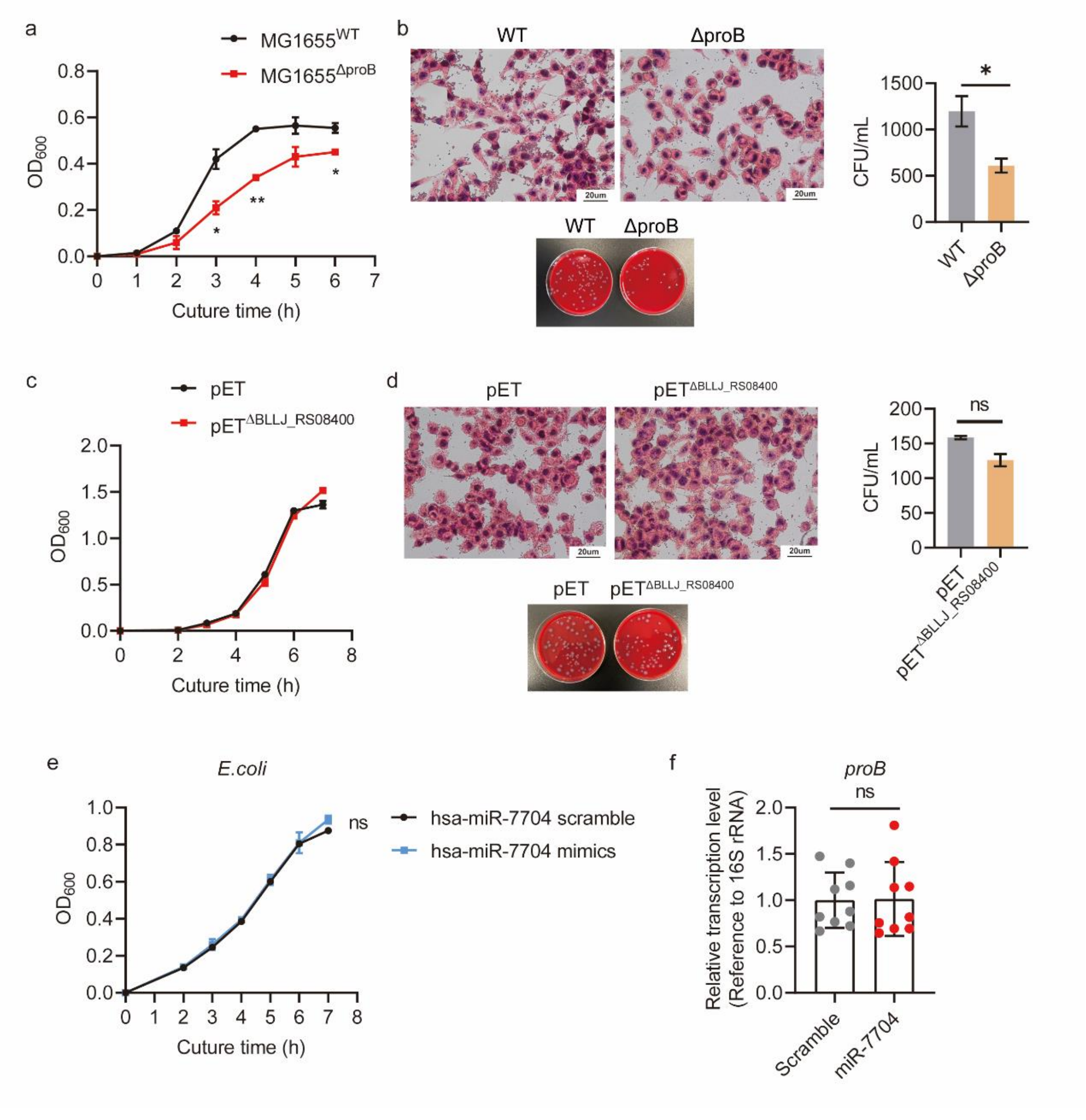

Supplementary Figure 4. Deletion of *proB* inhibited the growth and adhesion of *E. coli*. (a) Growth curve of *proB* deletion *E. coli* and control. (b) The representative images of HT-29 cells cultured with *proB* deletion *E. coli* and control. Gram staining. Scale bars, 20 μm. Quantification of adhesive bacteria. (c) Growth curve of BLLJ_RS08400 overexpression *E. coli* and control. (d) The representative images of HT-29 cells cultured with BLLJ_RS08400 overexpression *E. coli* and control. Gram staining. Scale bars, 20 μm. Quantification of adhesive bacteria. (e) Growth curve of *E. coli* cultured with 2 μM hsa-miR-7704 mimics or scramble. (f) Transcripts of *proB* at logarithmic phase were quantified by qPCR and normalized to 16S rRNA. Error bars denote mean ± SEM; two-tailed Student's t-test was performed,

*$p < 0.05$.

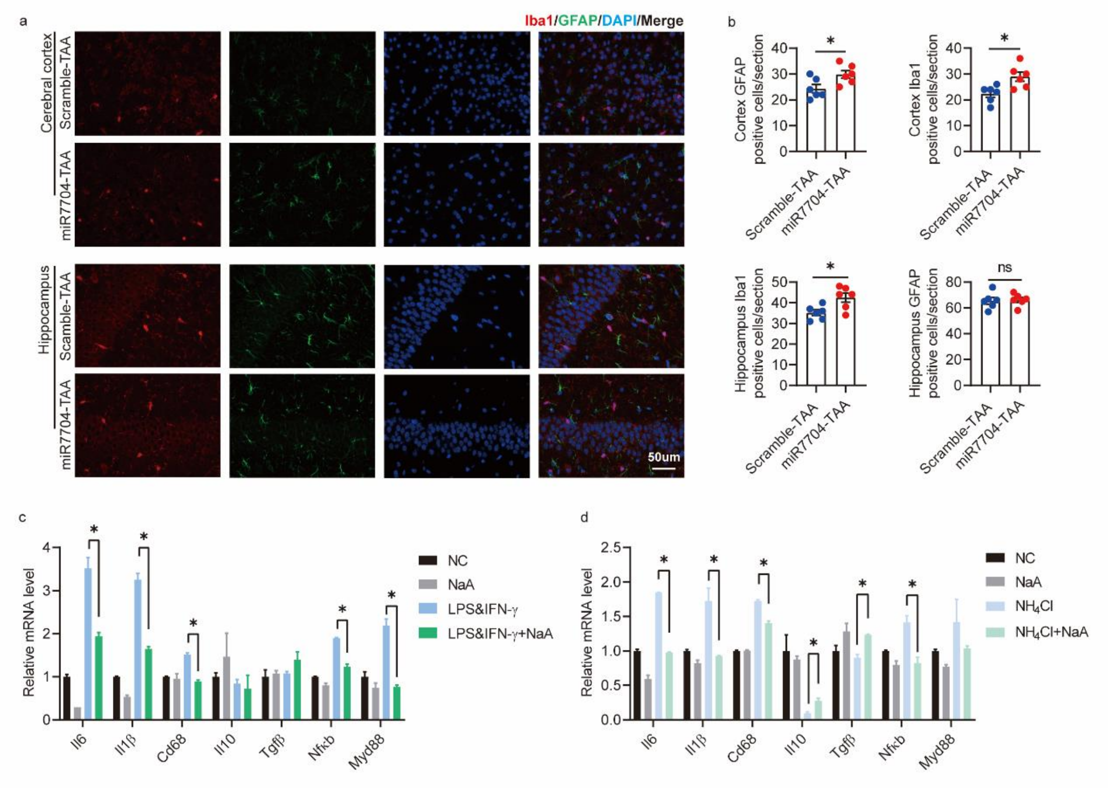

Supplementary Figure 5. Orally administration of hsa-miR-7704 promotes microglia activation. (a) Immunofluorescence staining of Iba1 and GFAP in the cerebral cortex and hippocampus; representative images are shown. Red: Iba1; green: GFAP; blue: DAPI. Scale bars, 50μm. (b) Statistics form of (a). (c and d) Relative gene expression of inflammatory cytokines. Human microglia cells (HMC3 cells) were treated with LPS＆IFN-γ (100ng/mL, 6h), NH_4Cl (10 mM, 24h) and NaA (5 mM, 6h). Data are represented as mean ± SEM and determined by two-way ANOVA or two-tailed Student's t-test. $*p < 0.05$.

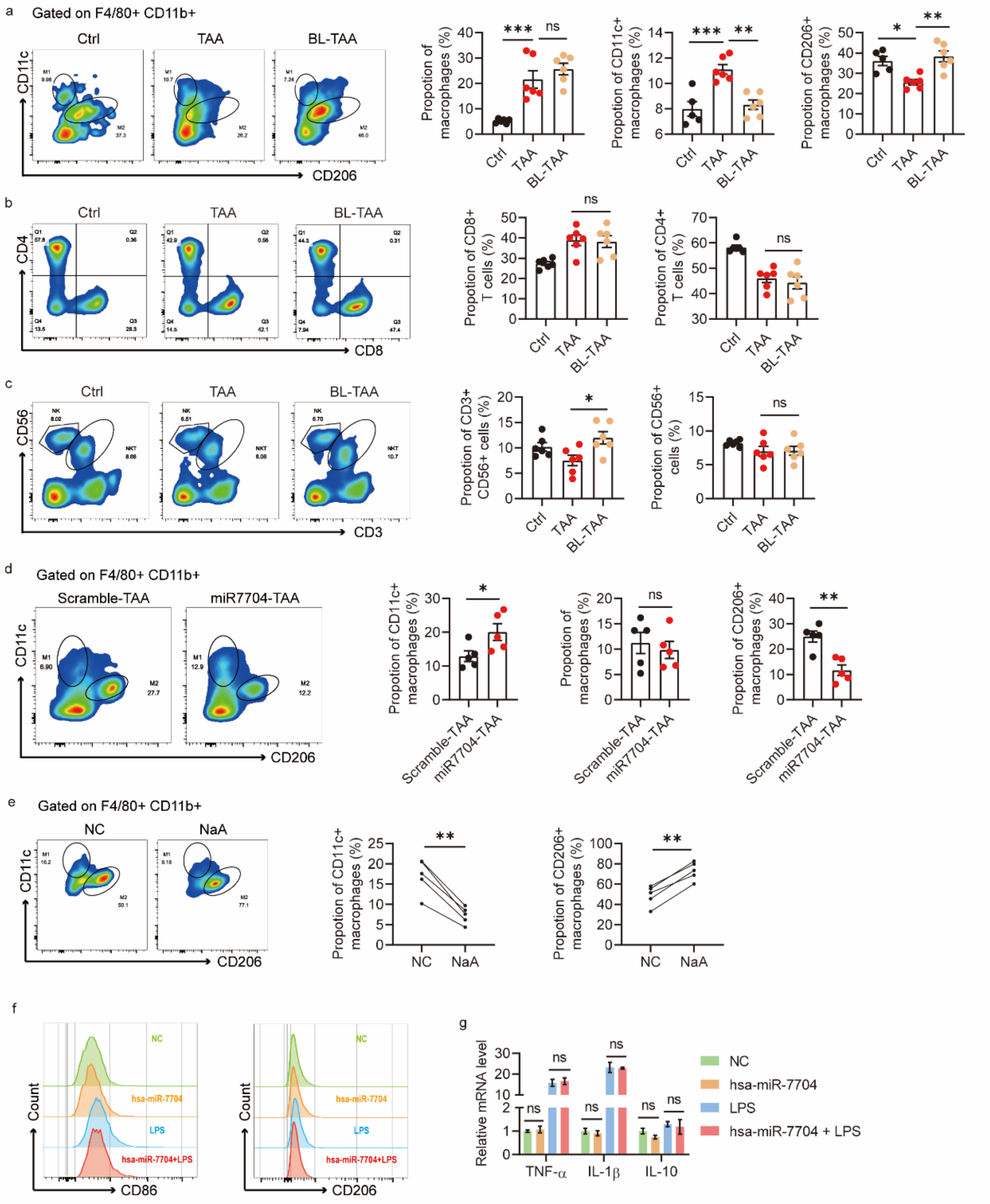

Supplementary Figure 6. *B. longum* and its metabolite acetate alleviate HE by promoting macrophages M2-type polarization (a-c) The effect of *B. longum* gavage on mice liver immune cells. 48 h after TAA induction, live immune cells were gated and determined for M1-type (CD11c+CD206-)/M2-type (CD206+CD11c-) macrophages, CD4+/CD8+ T cells, NK and NKT-like cells. (a) Representative FACS plots of liver M1-type/M2-type macrophages, gated on F4/80+ CD11b+. Statistics form were shown. (b) Representative FACS plots of liver CD4+/CD8+ T cells, gated on CD45+

CD3+. Statistics form were shown. (c) Representative FACS plots of liver NK and NKT-like cells. Statistics form were shown. (d) The effect of miR-7704 treatment on mice liver immune cells. Representative FACS plots of M1-type/M2-type macrophages, gated on F4/80+ CD11b+. Statistics form were shown. (e) Primary liver immune cells were separated from naïve mice and cultured with 10 mM acetate for 48 hours, then gated and determined for M1/M2-type macrophages. Cells from the same mouse were paired. Representative FACS plots of liver M1-type/M2-type macrophages, gated on F4/80+ CD11b+. Statistics form were shown. (f) The effects of hsa-miR-7704 on M1/M2 polarization of THP-1 cells. Representative FACS images are shown. (g) THP-1 cells were transfected with has-miR-7704 mimics, then treated with or without LPS (100ng/mL) for 6 hours. Transcription levels of TNF-α, IL-1β and IL-10 were detected by qPCR. Data are represented as mean ± SEM and determined by one-way ANOVA, unpaired or paired Student's t-test. *$p < 0.05$.

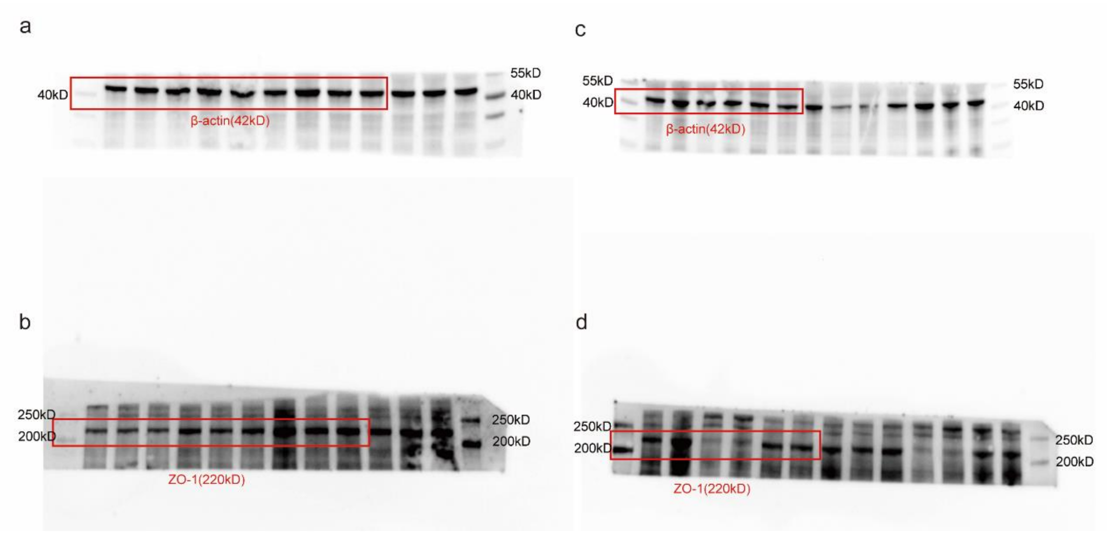

Supplementary Figure 7. Unprocessed gel images. (a-b) Unprocessed gel images for Fig 4d. a and b were derived from one gel. (c-d) Unprocessed gel images for Fig 6a. c and d were derived from one gel. The red box shows the blots that appear in the main text.
