# Supplementary material for: Faecal hsa-miR-7704 inhibits the growth and adhesion of Bifidobacterium longum by suppressing ProB and aggravates hepatic encephalopathy
Source: NPJ Biofilms Microbiomes. 2024 Feb 24;10:13. doi: 10.1038/s41522-024-00487-8 (PMC10891095; doi:10.1038/s41522-024-00487-8)
Supplement: Supplementary file 2 — Reporting-summary [file 41522_2024_487_MOESM2_ESM.pdf]

nature portfolio

Corresponding author(s): DAPR, 02628R

Last updated by author(s): Jan 10, 2024

Reporting Summary

Nature Portfolio wishes to improve the reproducibility of the work that we publish. This form provides structure for consistency and transparency in reporting. For further information on Nature Portfolio policies, see our Editorial Policies and the Editorial Policy Checklist.

Statistics

For all statistical analyses, confirm that the following items are present in the figure legend, table legend, main text, or Methods section.

n/a	Confirmed	
☐	☒	The exact sample size (*n*) for each experimental group/condition, given as a discrete number and unit of measurement
☐	☒	A statement on whether measurements were taken from distinct samples or whether the same sample was measured repeatedly
☐	☒	The statistical test(s) used AND whether they are one- or two-sided<br>*Only common tests should be described solely by name; describe more complex techniques in the Methods section.*
☒	☐	A description of all covariates tested
☒	☐	A description of any assumptions or corrections, such as tests of normality and adjustment for multiple comparisons
☐	☒	A full description of the statistical parameters including central tendency (e.g. means) or other basic estimates (e.g. regression coefficient) AND variation (e.g. standard deviation) or associated estimates of uncertainty (e.g. confidence intervals)
☐	☒	For null hypothesis testing, the test statistic (e.g. *F*, *t*, *r*) with confidence intervals, effect sizes, degrees of freedom and *P* value noted<br>*Give P values as exact values whenever suitable.*
☒	☐	For Bayesian analysis, information on the choice of priors and Markov chain Monte Carlo settings
☒	☐	For hierarchical and complex designs, identification of the appropriate level for tests and full reporting of outcomes
☐	☒	Estimates of effect sizes (e.g. Cohen's *d*, Pearson's *r*), indicating how they were calculated

Our web collection on statistics for biologists contains articles on many of the points above.

Software and code

Policy information about availability of computer code

Data collection	Metagenomic data were collected by Illumina NovaSeq. 16S rRNA data were sequenced on NovaSeq PE250.
Data analysis	All data are analyzed with GraphPad Prism.

For manuscripts utilizing custom algorithms or software that are central to the research but not yet described in published literature, software must be made available to editors and reviewers. We strongly encourage code deposition in a community repository (e.g. GitHub). See the Nature Portfolio guidelines for submitting code & software for further information.

Data

Policy information about availability of data

All manuscripts must include a data availability statement. This statement should provide the following information, where applicable:

- Accession codes, unique identifiers, or web links for publicly available datasets
- A description of any restrictions on data availability
- For clinical datasets or third party data, please ensure that the statement adheres to our policy

The metagenomic sequencing data, 16S rRNA data, and miRNA microarray data used in this study are openly available in NCBI (PRJNA953603) and GEO (GSE228731; GSE228827).

Research involving human participants, their data, or biological material

Policy information about studies with human participants or human data. See also policy information about sex, gender (identity/presentation), and sexual orientation and race, ethnicity and racism.

Reporting on sex and gender	Human fecal specimens were collected from 10 CHB subjects (8 males, average 51 years of age) and 9 HE subjects (7 males, average 54 years of age).
Reporting on race, ethnicity, or other socially relevant groupings	All subjects were excluded for cancers, diabetes, alcoholic liver diseases or COVID-19. A patient who received antibiotics within the last two months and during the sampling period was excluded from the study.
Population characteristics	10 CHB subjects and 9 HE subjects
Recruitment	In order to collect stool samples, Fisher Scientific's Commode specimen collection system was used, and the samples were stored at -80°C until further processing was carried out.
Ethics oversight	A protocol approved by the Clinical Research Ethics Committee of the First Affiliated Hospital, Zhejiang University School of Medicine, was followed by all subjects (NO. 2021-029).

Note that full information on the approval of the study protocol must also be provided in the manuscript.

Field-specific reporting

Please select the one below that is the best fit for your research. If you are not sure, read the appropriate sections before making your selection.

☒ Life sciences ☐ Behavioural & social sciences ☐ Ecological, evolutionary & environmental sciences

For a reference copy of the document with all sections, see nature.com/documents/nr-reporting-summary-flat.pdf

Life sciences study design

All studies must disclose on these points even when the disclosure is negative.

Sample size	There were no statistical methods to predetermine sample size. For animal studies, the sample size of the animal experiments was at least 5 or more animals in order to obtain statistical significance.
Data exclusions	One metagenomic data was culled because of deviations from the group average.
Replication	All data representative of two or more independent experiments.
Randomization	The animals were randomly allocated into different groups, and the average body weights for each group have to be statistically not significant before the treatments.
Blinding	Investigators were blinded to group allocation during data collection and analysis. But blinding is not possible for every experiments, during the treatment of live animals it was not blinded, as the treatment of each mouse would need to be known to the person handling the mice.

Reporting for specific materials, systems and methods

We require information from authors about some types of materials, experimental systems and methods used in many studies. Here, indicate whether each material, system or method listed is relevant to your study. If you are not sure if a list item applies to your research, read the appropriate section before selecting a response.

Materials & experimental systems

n/a	Involved in the study	
☐	☒	Antibodies
☒	☐	Eukaryotic cell lines
☒	☐	Palaeontology and archaeology
☐	☒	Animals and other organisms
☒	☐	Clinical data
☒	☐	Dual use research of concern
☒	☐	Plants

Methods

n/a	Involved in the study	
☒	☐	ChIP-seq
☐	☒	Flow cytometry
☒	☐	MRI-based neuroimaging

Antibodies

Antibodies used	APC-Cy7-anti-Mouse F4/80, Pacific Blue-anti-Mouse CD11b, FITC-anti-Mouse CD11c, APC-anti-Mouse CD206, APC-Cy7-anti-Mouse TCRβ, APC-anti-Mouse NK1.1, PerCP-Cy5-5-anti-Mouse CD45, PE-Cy7-anti-Mouse CD3, PE-anti-Mouse CD4, FITC-anti-Mouse CD8, APC-anti-Mouse TNF-α, all purchased from Biolegend; FITC-anti-Human CD86 (20 µl/ test), APC-anti-Human CD206 (20 µl/test), purchased from BD Pharmingen.
Validation	All the antibodies were commercially available and their validation statements are available on the manufacturers' websites. All antibodies were validated as per manufacturers' instructions.

Animals and other research organisms

Policy information about studies involving animals; ARRIVE guidelines recommended for reporting animal research, and Sex and Gender in Research

Laboratory animals	6-10 weeks old male C57BL/6 J mice were purchased from the Shanghai SLAC Laboratory Animal Co.,Ltd
Wild animals	This study did not involve wild animals.
Reporting on sex	The mice used in this experiment were all male. Hepatic encephalopathy is more common in men.
Field-collected samples	Field-collected samples were not used in this study.
Ethics oversight	All animal experimental procedures were approved by Research Ethics Committee of the First Affiliated Hospital, College of Medicine, Zhejiang University (NO. 2021-07).

Note that full information on the approval of the study protocol must also be provided in the manuscript.

Plants

Seed stocks	*Report on the source of all seed stocks or other plant material used. If applicable, state the seed stock centre and catalogue number. If plant specimens were collected from the field, describe the collection location, date and sampling procedures.*
Novel plant genotypes	*Describe the methods by which all novel plant genotypes were produced. This includes those generated by transgenic approaches, gene editing, chemical/radiation-based mutagenesis and hybridization. For transgenic lines, describe the transformation method, the number of independent lines analyzed and the generation upon which experiments were performed. For gene-edited lines, describe the editor used, the endogenous sequence targeted for editing, the targeting guide RNA sequence (if applicable) and how the editor was applied.*
Authentication	*Describe any authentication procedures for each seed stock used or novel genotype generated. Describe any experiments used to assess the effect of a mutation and, where applicable, how potential secondary effects (e.g. second site T-DNA insertions, mosiacism, off-target gene editing) were examined.*

Flow Cytometry

Plots

Confirm that:

☒ The axis labels state the marker and fluorochrome used (e.g. CD4-FITC).

☒ The axis scales are clearly visible. Include numbers along axes only for bottom left plot of group (a 'group' is an analysis of identical markers).

☒ All plots are contour plots with outliers or pseudocolor plots.

☒ A numerical value for number of cells or percentage (with statistics) is provided.

Methodology

Sample preparation	For surface staining, the cell suspension was incubated with fluorescently labeled antibody at room temperature for 20 minutes. For CD206 staining, the samples were fixed and permeabilized with BD Cytofix/Cytoperm Fixation/Permeabilization Solution kit (BD Biosciences, US), and then incubated with fluorescently labeled antibody at 4°C for 35 minutes. For intracellular TNF-α staining, the samples were stimulated by LPS (100 ng/ml, Beyotime) for 4 h, fixed and permeabilized, and then incubated with fluorescently labeled antibody at 4°C for 35 minutes. Bacteria were stained using the BacLight™ Red kit (Invitrogen, B-35001). miRNA was labelled with fluorescein amidites (FAM).
Instrument	Flow cytometry or Image Stream was conducted using a BD FACS CantoII or Millipore ISX with fluorochrome-conjugated cells.
Software	Data was analyzed using FlowJo software version 10.4 or IDEAS version 6.0.

Cell population abundance	Cell populations and mean fluorescence intensity were calculated by FlowJo software.
Gating strategy	Macrophages were gated by F4/80+ CD11b+. Under this gate, M1-type were gated by CD11c+ CD206-, while M2-type were gated by CD206+ CD11c-.

☒ Tick this box to confirm that a figure exemplifying the gating strategy is provided in the Supplementary Information.
